# Application of feature-based molecular networking and MassQL for the MS/MS fragmentation study of depsipeptides

**DOI:** 10.3389/fmolb.2023.1238475

**Published:** 2023-08-01

**Authors:** Denise M. Selegato, Ana C. Zanatta, Alan C. Pilon, Juvenal H. Veloso, Ian Castro-Gamboa

**Affiliations:** ^1^ Nucleus of Bioassays, Biosynthesis, and Ecophysiology of Natural Products (NuBBE), Institute of Chemistry, São Paulo State University (UNESP), Araraquara, Brazil; ^2^ Núcleo de Pesquisa em Produtos Naturais e Sintéticos (NPPNS), Faculdade de Ciências Farmacêuticas, São Paulo University (USP), São Paulo, Brazil

**Keywords:** MS/MS fragmentation, beauvericin, feature-based molecular networking, MassQL, PCA

## Abstract

The Feature-based Molecular Networking (FBMN) is a well-known approach for mapping and identifying structures and analogues. However, in the absence of prior knowledge about the molecular class, assessing specific fragments and clusters requires time-consuming manual validation. This study demonstrates that combining FBMN and Mass Spec Query Language (MassQL) is an effective strategy for accelerating the decoding mass fragmentation pathways and identifying molecules with comparable fragmentation patterns, such as beauvericin and its analogues. To accomplish this objective, a spectral similarity network was built from ESI-MS/MS experiments of *Fusarium oxysporum* at various collision energies (CIDs) and paired with a MassQL search query for conserved beauvericin ions. FBMN analysis revealed that sodiated and protonated ions clustered differently, with sodiated adducts needing more collision energy and exhibiting a distinct fragmentation pattern. Based on this distinction, two sets of particular fragments were discovered for the identification of these hexadepsipeptides: ([M + H]^+^) *m/z* 134, 244, 262, and 362 and ([M + Na]^+^) *m/z* 266, 284 and 384. By using these fragments, MassQL accurately found other analogues of the same molecular class and annotated beauvericins that were not classified by FBMN alone. Furthermore, FBMN analysis of sodiated beauvericins at 70 eV revealed subclasses with distinct amino acid residues, allowing distinction between beauvericins (beauvericin and beauvericin D) and two previously unknown structural isomers with an unusual methionine sulfoxide residue. In summary, our integrated method revealed correlations between adduct types and fragmentation patterns, facilitated the detection of beauvericin clusters, including known and novel analogues, and allowed for the differentiation between structural isomers.

## 1 Introduction

LC-MS/MS-based metabolomics is a well-established technique for analyzing metabolites present in biological systems. However, the process of metabolite identification remains challenging. The structural complexity, and presence of similar fragments across different compounds, stereoisomers, adducts, and other spectral interferences make metabolite annotation a difficult task, even for experienced spectroscopists. Consequently, a significant number of metabolites in biological systems remain unidentified (de Jonge et al., 2023).

Although statistical tools have been developed for analyzing large datasets, they are primarily designed for data exploration and analysis rather than the specific task of metabolite annotation. These tools often rely on clustering, classification, or regression approaches, which may not provide the level of detail required for accurate and comprehensive metabolite identification. Therefore, specialized approaches are needed to effectively address the challenges associated with metabolite annotation in LC-MS-based metabolomics (Lindon et al., 2000; Vuckovic, 2012; Johnson et al., 2016; Wishart, 2016).

In recent years, molecular networking has emerged as a powerful tool for large-scale annotation of LC-MS/MS data (Wang et al., 2016). Molecular networking groups the mass spectra (MS^2^) of metabolites in a dataset based on fragmentation patterns, such as neutral losses, comparable losses, and ions present in the mass spectra. This approach offers a systematic way to organize and analyze vast amounts of LC-MS/MS data, allowing for the identification and classification of metabolites into distinct metabolic classes (Pilon et al., 2019). This network can also be enriched by annotating the experimental MS^2^ spectra against MS^2^ spectral libraries or compound databases, propagating annotations through the network edges to adjacent unknown nodes (Schmid et al., 2021).

Several investigations have demonstrated the effectiveness of molecular networking for metabolite annotation. For example, Naman et al. (2017) successfully identified a novel cytotoxic peptide from *Symploca* sp. cyanobacteria using molecular networks and the GNPS library (Naman et al., 2017). Similarly, Klein-Júnior et al. (2017) analyzed *Palicourea sessilis* using molecular networks and the DNP-ISDB tool, resulting in the identification of eight monoterpene alkaloids, three of which were novel and exhibited moderate anticholinesterase activity (Klein-Júnior et al., 2017). Olivon et al. (2017) employed molecular networks and bioactivity data against the Chikungunya virus to prioritize and isolate four esters of 12-deoxyforbol, two of which were novel, from 107 Euphorbiaceae species with anti-viral activity (Olivon et al., 2017).

Molecular networks have also been utilized to discover chalcones with antimicrobial properties against *Staphylococcus aureus* from *Angelica keiskei* (Caesar et al., 2018), as well as to characterize new indole alkaloids from *Geissospermum laeve* with potential antiparasitic and cytotoxic activity (Fox Ramos et al., 2017). Furthermore, Nothias et al. (2018) developed the bioactive molecular network approach for bio-guided studies, facilitating the dereplication process by associating bioactivity values with ion quantifications detected in LC-MS experiments (Nothias et al., 2018). This approach led to the isolation of two new substances, maridric acids A and B, from previously uncharacterized marine microorganisms.

The use of molecular networking can also assist in understanding metabolic fragmentation. This method involves analyzing metabolites with different collision energies, resulting in unique cleavage patterns. By examining protonation sites, cleavage types, fragment stability, and characteristic ions, researchers can accurately identify metabolites and their specific fragmentation patterns. Importantly, this approach would reveal the connections between structurally similar metabolites by highlighting subtle and intricate fragmentation patterns (De Souza et al., 2020; de Jonge et al., 2023).

Based on advancements in molecular networking, Jarmusch et al. (2022) have developed a complementary method called MassQL to further explore underutilized MS/MS data (Jarmusch et al., 2022). MassQL captures the unique characteristics of MS data, including isotopic patterns, diagnostic fragmentation, and neutral loss, and establishes a comprehensive MS terminology for searching MS patterns across datasets. This powerful tool formalizes terms for MS1 patterns (e.g., precursor ion *m/z*, isotopic patterns) and MS/MS fragmentation patterns, ensuring compatibility with all types of mass spectrometry data. By integrating molecular networking with MassQL, researchers can employ a comprehensive and powerful approach to analyze LC-MS/MS data and gain insights into metabolite fragmentation pathways.

In the present study, our aim is to demonstrate the potential of combining principal component analysis (PCA), spectral similarity networking, and MassQL as an effective approach for decoding mass fragmentation pathways of beauvericins and analogues. The beauvericin class a cyclic hexadepsipeptides composed of alternating *N*-methyl amino acid and hydroxy acid residues (Urbaniak et al., 2020). This class shares structural similarities with other enniantins but differs primarily in the types of amino acids and hydroxy acids that encompass them. Enniantins usually contain 2-hydroxyisovaleric acid, *N*-methylvaline, *N*-methylisoleucine, *N*-methylleucine (Sy-Cordero et al., 2012; Renaud et al., 2017; Li et al., 2020), while beauvericins commonly feature *N*-methylpheylalanine (MePhe), 2-hydroxyisovaleric acid (Hiv) and 2-hydroxy-3-methylpentanoic acid (Hamill et al., 1969; Gupta et al., 1995; Li et al., 2020; Urbaniak et al., 2020).

Due to similar fragmentation of peptides and the challenge to identify them, the beauvericins were selected as a model metabolic class for application of our strategy using FBMN and MassQL. Depsipeptides usually have the proton located at the *N*-terminus or a basic residue side chain and, during fragmentation, it can move along the backbone, breaking at different conserved sites (Pallerla et al., 2019). Thus, to achieve this objective, we employ these approaches in a combination of different collision energies from ESI-MS/MS experiments from a *Fusarium oxysporum* extract to determine both the mass fragmentation pathways and the identification of known and novel hexadepsipeptides. The selection of these compound classes is based on the ongoing debate surrounding the fragmentation profile of beauvericin, specifically the assignment of the ion at *m/z* 362 (Liuzzi et al., 2017; Tolosa et al., 2019). While this ion is commonly identified as diagnostic fragment ions for beauvericin and some derivatives, there is controversy regarding their presence in other cyclic peptides, lacking the required specificity for their automated discovery. Thus, our study aims to provide insights into the ambiguous fragmentation between these compound classes and demonstrate the potential of our approach in deciphering these complex fragmentation patterns.

## 2 Materials and methods

### 2.1 Fungi fermentation and metabolite extraction

*Fusarium oxysporum* (Nectriaceae) was cultured in Czapek-Broth (NaNO_3_, 1.5 g L^−1^; KH_2_PO_4_, 0.5 g L^−1^; MgSO_4_, 0.25 g L^−1^; FeSO_4_.7H_2_O, 0.025 g L^−1^; KCl, 2.5 g L^−1^; and D-glucose, 30.0 g L^−1^) in three biological replicates. Each fungal replicate was cultivated separately in eight Erlenmeyer flasks containing 300 mL of Czapek broth. The medium was first autoclaved at 121 °C for 20 min and, after sterilization, *F. oxysporum* was inoculated and incubated while stationary at 26°C for 28 days.

At the end of the incubation period, the flasks were vacuum filtered to remove mycelium and extracted with ethyl acetate (3 × 500 mL). The solvent was evaporated, and the extracts were then submitted to a clean-up process with solid phase extraction (SPE) in a cartridge filled with C-18 reversed phase silica after reconstitution in methanol HPLC grade (Strata X, C18), followed by filtration in 0.22 µM membrane.

### 2.2 MS/MS parameter optimization

Direct flow infusion of the samples was performed using a high-resolution micrOTOF-QII mass spectrometer (Bruker Daltonics, Bremen, Germany) for the optimization of the ionization and fragmentation parameters. This preliminary analysis used beauvericin as targeted precursor ions, both in its protonated (theoretical *m/z* 784.4173) and sodiated (*m/z* 806.3992) adducts, aiming to obtain MS/MS data with distinctive product ions characteristic. To optimize the collision energy (CE), fragmentation experiments were performed altering the collision-induced dissociation (CID) energy. For each experiment, these precursor ions were selected from the full-scan mass spectrum using a mass error of 10 ppm and fragmented at different CID energies (10, 20, 25, 30, 40, 50, 60 and 70 eV). Optimized CID energies were selected based on the ability to produce different MS/MS spectra in terms of type of product ions and their intensity.

The optimized ESI parameters were set to the following values: positive ionization mode, nebulizer (N_2_) gas pressure of 4.5 Bar, dry gas flow rate of 9.0 L min^-1^, ion source temperature of 200°C, capillary voltage of 4500 V, and voltage source of 5 kV. Full scan analysis was conducted in the *m/z* range of 50–1,500.

### 2.3 LC-MS/MS parameters

The LC-MS/MS analysis of the extracts of each replicate of *F. oxysporum* was performed using ultra-fast liquid chromatography (UFLC) Shimadzu system (Shimadzu Prominence UFLC, Shimadzu) equipped with two solvent pumps (LC-20AD), a degassing system (DGU-20A3), an autosampler (SIL-20AHT), a column oven (CTO-20A), a system controller (CBM-20A), and a diode array detector (SPD-M20AV, Shimadzu). The UFLC system was coupled to the previously described high-resolution micrOTOF-QII mass spectrometer.

To perform the analysis, the samples were suspended in MeOH/H_2_O (8:2, v/v) at a concentration of 5.0 mg mL^-1^, centrifuged, and the supernatant was transferred to a vial for subsequent analysis. LC separations were performed on a Kinetex 2.6 μm XB-C18 core-shell column (100 × 2.1 mm ID, Phenomenex, Torrance, United States). The injection volume was 2 μL, the flow rate was 250 μL min^-1^, and the column oven temperature was set to 40°C. The samples were eluted using a mobile phase consisting of water (solvent A) and acetonitrile (solvent B), both acidified with 0.1% formic acid, in a linear gradient from 5% to 100% (B) over a period of 45 min.

ESI and MS/MS conditions were set based on the previously optimized parameters. The spectra acquisition rate was established at 1 Hz. The instrument provided a resolving power of 9,000 per FWHM for the beauvericin precursor ion *m/z* 784.4173. The mass spectra were externally calibrated in Enhanced Quadratic mode, using the exact masses of the sodium trifluoroacetate (NaTFA) clusters ions from a 500 ppm solution in methanol/water (1:1, v/v). After each run, the calibrant solution was consistently injected at a flow rate of 3 μL/min via a six-port divert valve. The MS/MS fragmentation step involved selecting the 5 ions with the highest intensity, and active exclusion was applied after 4 spectra with a release time of 30 s. The mass spectra were processed using the DataAnalysis software (version 4.3, Bruker). MS data collected from *F. oxysporum* replicates can be found in the MassIVE dataset under the register number MSV000091616.

### 2.4 LC-MS/MS data processing

The mass spectrometry data were centroided and converted from the proprietary format (.raw) to the *m*/*z* extensible markup language format (.mzML) using the peak picking algorithm of ProteoWizard (ver. 3.0.19, MSConvert tool) (Chambers et al., 2012). The mzML files were then processed with MZmine3 (Schmid et al., 2023). In short, feature detection and deconvolution were performed with the ADAP chromatogram builder and local minimum resolver algorithm. The isotopologues were regrouped and the features were aligned, and gap filled across samples.

The aligned peak list was filtered to contain only peaks with an associated fragmentation spectrum. Finally, the feature quantification table results (.CSV) and spectral information (.MGF) were exported with the GNPS module for feature-based molecular networking analysis on GNPS and with SIRIUS export modules. The MZmine3 project, the MZmine3 batch file (.XML format), and results files (.MGF and. CSV) are available in the MassIVE dataset under the register number MSV000091616. The MZmine3 batch file contains all the parameters used during the processing.

### 2.5 LC–MS/MS data annotation

The files exported from MZmine3 were uploaded to GNPS (http://gnps.ucsd.edu) (Wang et al., 2016) platform, in which spectral library matching was performed against public fragmentation spectra (MS2) spectral libraries. Annotation has also been performed with SIRIUS (v. 5.5.7) to systematically annotate the MS2 spectra (Böcker et al., 2009; Böcker and Dührkop, 2016; Dührkop et al., 2019; 2021). Molecular formulas were computed with the SIRIUS module by matching the experimental and predicted isotopic patterns and from fragmentation trees analysis of MS2. The parameters for SIRIUS tools were set as follows: molecular formula candidates retained, 10; maximum precursor ion *m*/*z* computed, 850; profile, Q-TOF; MS2 mass accuracy (ppm), 10; possible ionizations [M + H]^+^ and [M + Na]^+^.

### 2.6 Feature-based molecular networking

A molecular network was created with the FBMN workflow (Nothias et al., 2020) on GNPS (Wang et al., 2016). The precursor ion mass tolerance was set to 0.01 Da and the MS/MS fragment ion tolerance to 0.02 Da. A molecular network was then created where edges were filtered to have a cosine score above 0.7 and more than 4 matched peaks. Further, edges between two nodes were kept in the network if and only if each of the nodes appeared in each other’s respective top 10 most similar nodes. Finally, the maximum size of a molecular family was set to 100, and the lowest-scoring edges were removed from molecular families until the molecular family size was below this threshold.

The analogue search mode was used by searching against MS/MS spectra with a maximum difference of 100.0 in the precursor ion value. The DEREPLICATOR software was used to annotate MS/MS spectra (Mohimani et al., 2018). The molecular networks were visualized using the Cytoscape software (Shannon et al., 2003) and the links are available in the Supplementary Materials for public access.

### 2.7 Principal component analysis (PCA)

The.MGF file containing the MS/MS information was binned (bin size of *m/z* 0.1) and the resulting table was used to visualize the clustering of compounds based on the presence of conserved ions using Principal Component Analysis. SDV function from the R package (version 2.5.5) was applied to calculate the Euclidean dissimilarity matrix based on the metabolite levels. Subsequently, classical metric multidimensional scaling was carried out based on the Euclidean distance matrix to obtain different principal coordinates.

### 2.8 MassQL analysis

The MassQL tool version 31.4 (Jarmusch et al., 2022) was employed to search for specific MS/MS fragments that were identified as specific for the beauvericin molecular class. Thus, these analogs were grouped by searching for MS/MS diagnostic ions at (1) *m/z* 134, 244, 262, 362 or (2) *m/z* 384, 284, 266 with a 0.1 m*/z* tolerance and a minimum percentage intensity relative to the base peak of 10.0% for analysis at CID energy of 25 eV, and 100% for 50 and 70 eV. The MassQL jobs can be publicly accessed, and the links are available in the Supplementary Materials. The extracted data was re-analyzed to generate a molecular network, using the same parameters as described above.

## 3 Results

To assess the ability of MS/MS Molecular Networking to detect beauvericin analogues and distinguish their fragmentation patterns in different collision energies (25, 50 and 70 eV), a fragmentation study was established by the untargeted tandem mass spectrometry of *F. oxysporum* extract based on the fast data-independent acquisition (DIA) function of QToF mass spectrometer. This *Fusarium* extract has been previously reported to produce beauvericin (Selegato et al., 2016) and the DIA setting allowed the automatic analysis and detection of known and unknown analogs that range in structural features and concentration in these fungal cultures.

### 3.1 Principal component analysis (PCA) of MS/MS data

**FIGURE 1:**
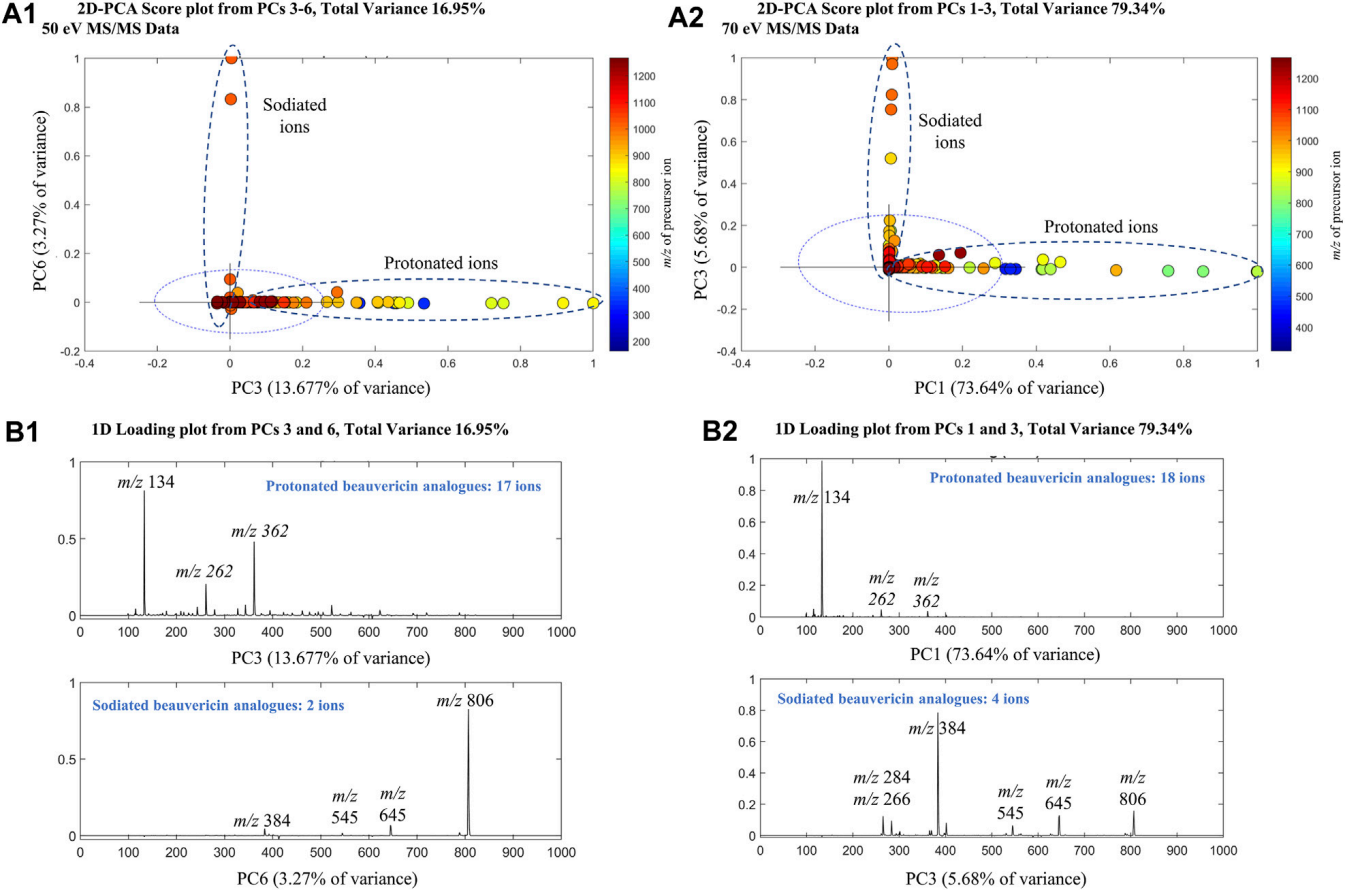
Principal Component Analysis (PCA) of the MS/MS beauvericin data at 50 eV and 70 eV. Analysis was performed using tandem MS data at different collision energies to obtain principal components (PCs) that explained the total beauvericin variation **(A)** 2D-Score plots of the PCs for 50 (PC3 and PC6) and 70 eV (PC1 and PC3). **(B)** 1D-Loading plots of the PCs that displayed a strong correlation was observed with beauvericin. For 50 eV, PC3 and PC6 show a combined variance of 16.95%, whereas for 70eV, PC1 and PC3 have 79.34% of explained variance.

PCA analysis was performed on the MS/MS data using the SDV algorithm to evaluate the effect of collision energy on fragmentation data of beauvericin and analogues. Separate PCAs were conducted for each collision energy, showing a cumulative explained variance of 58.27% (25 eV), 70.42% (50 eV), and 96.32% (70 eV) for the first five principal components (PCs). For the higher collision energy, a strong correlation was observed between beauvericin in PCs 3–6 (for 50 eV) and PCs 1–3 (for 70 eV), respectively (Figure 1). However, this correlation was associated with the type of adduct formed during MS acquisition (protonated and sodiated ions) rather than the fragmentation pattern itself.

The protonated beauvericins [M + H]^+^ were clustered on the positive sides of PC3 and PC1 (for 50 and 70 eV, respectively) with four characteristic fragments identified: *m/z* 362, 262, 244 and 134. The *m/z* 134 fragment exhibited higher abundance at 70 eV and corresponds to the *N*-methylphenylalanine (MePhe) residue, while the ions *m/z* 244, 262, and 362 showed higher intensities at 50 eV and are associated with the c2 and b2-fragments of MePhe-Hiv residues and the b3-fragment of the Hiv-MePhe-Hiv residue, respectively. In general, the depsipeptide fragmentation occurs mostly at the peptide bond (b-fragments), leading to the formation of stable amino acid immonium ions (H_2_N^+^ = CHR_2_), or, at higher energies, in neighboring sites, generating the a-fragment alkyl carbonyl (CHR-CO) and the c-fragment aminoalkyl bond (NH-CHR) (Hohmann et al., 2008).

Contrarily, the positive sides of PC6 and PC3 (for 50 and 70 eV, respectively) were dominated by sodiated adducts [M + Na]^+^ which are clustered due to the presence of the ions *m/z* 384, 284, and 266. These fragments are also originated from the b3-fragmentation of Hiv-MePhe-Hiv residue and the b2/c2-fragments of the MePhe-Hiv residue, respectively. Interestingly, the sodiated adducts exhibited a lower overall abundance of fragments compared to the protonated ions, indicating increased stability of the Na^+^ adducts. Only the ion *m/z* 384 was significant at both 50 and 70 eV, while no fragments were observed for beauvericin at 25 eV. Lastly, a higher number of beauvericin ions were clustered at 70 eV, confirming that at higher energy levels, the conserved ions are more prevalent and explain the variance in the dataset better than other fragments and the precursor ion.

PCA analysis mainly showed the significant distinctions between sodiated and protonated beauvericin adducts. However, evaluation of the diagnostic fragments correlated with this adducts also reveals variations in the fragmentation pattern, specifically regarding the type and quantity of fragments generated through peptide cleavages. Indeed, the representative fragmentation spectra of beauvericin suggests that lower energies break the protonated ions into both b and c fragments, along with neutral loss of water (−18 Da) and methyl (−15 Da), whereas at higher energies, more stable b2-fragments have higher abundance in the spectra.

**FIGURE 2:**
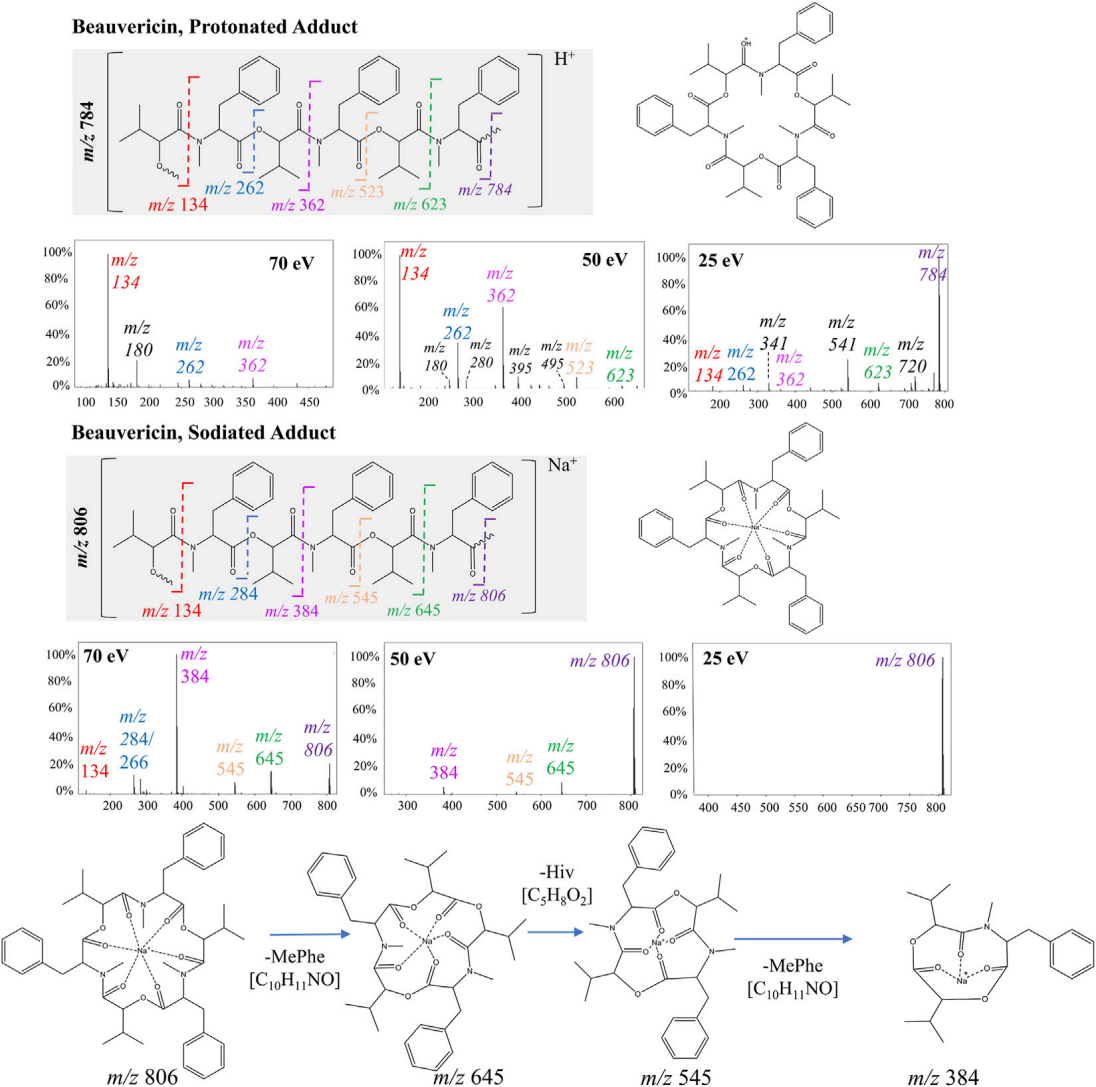
Fragmentation of beauvericin as a protonated ion and sodiated adduct at 25, 50, and 70 eV. The structure is shown both in its linear and cyclic form to facilitate visualization. Breaks in the peptide bond of each amino acid residue are shown in different colors and correspond to the b-fragments.

For protonated beauvericin, the most abundant fragment at 25 eV is the c4-fragment at *m/z* 541, as well as all five b-fragments (*m/z* 134, 262, 362, 523 and 623). As the collision energy increases, these b-fragments become more dominant over the c-fragments, in which the most significant ions are one, two and three-residue b-fragments (*m/z* 134, 262 and 362). At 70 eV, most fragments above *m/z* 400 have undergone fragmentation, and the *m/z* 262 and 362 appear at low intensities. Consequently, the single residues MePhe (*m/z* 134) and OH-Hiv (*m/z* 180) become the only representative fragments, with normalizes abundances of 100% and 20%, respectively (Figure 2). This fragmentation plurality is not observed in sodiated adducts, regardless of the collision energy. At 25 eV, no fragments are generated, and, at 50 and 70 eV, only b-fragments are observed. Interestingly, neither a nor c-fragments were formed at any of the collision energies, resulting in a cleaner spectrum compared to their respective protonated precursor.

### 3.2 Feature-based molecular networking (FBMN) of beauvericin analogues in different collision energies

The FBMN approach involves organizing MS/MS data (spectra) into a network based on the similarity of fragmentation patterns. Each node within the network represents a collection of spectra sharing the same precursor ion, and the relationships between nodes are determined by the degree of similarity among the spectra. Thus, to examine the variations in the fragmentation pattern of beauvericins under different CID energies, each dataset (25, 50, and 70 eV) was submitted to molecular networking approach.

**FIGURE 3:**
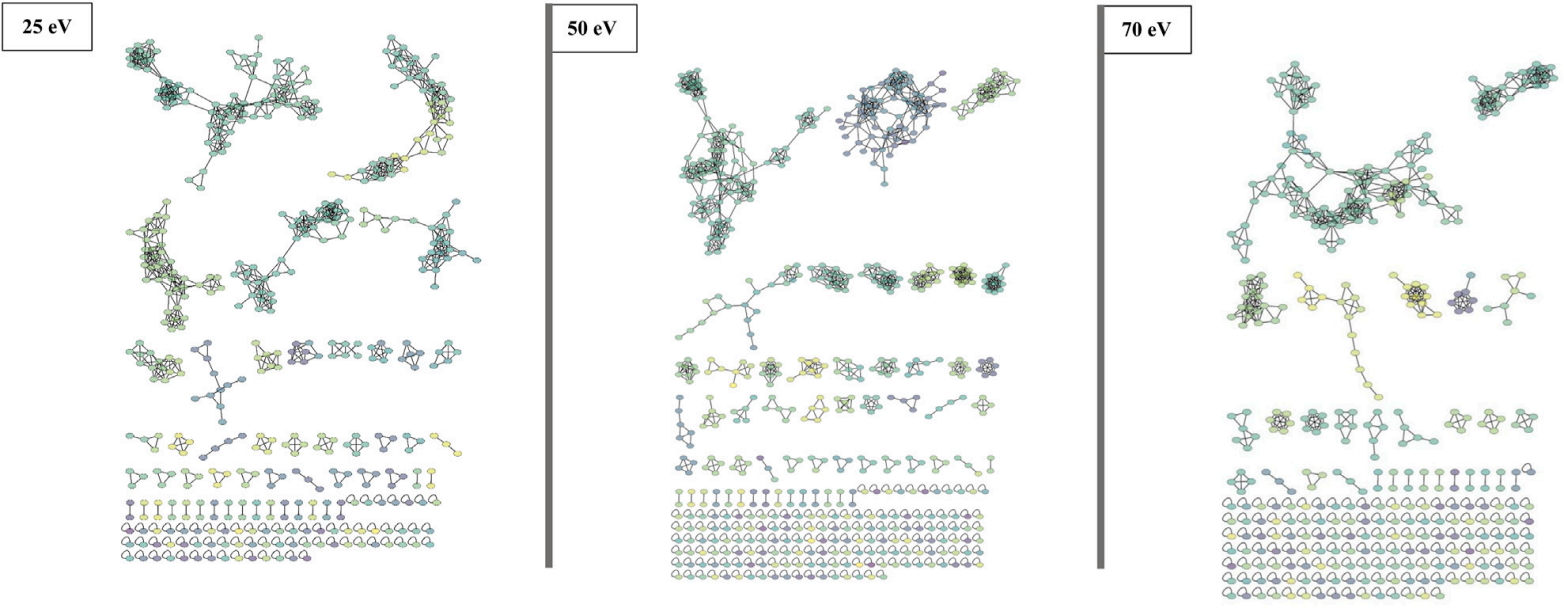
FBMN of beauvercin analogues at different collision energies (25, 50, and 70 eV). Each dataset was obtained from replicates of the same fungi extract and contains a similar number of MS/MS features. Nodes are colored according to the mass of the parent ions, in which yellowish colors represent lower masses (∼*m/z* 250) and purple-like nodes constitute higher masses (∼*m/z* 1,200).

Although all three networks contain a similar number of precursor ions, there is a disparity in the number of clusters. At higher collision energy (70 eV), there was a higher prevalence of singletons, which do not exhibit similar fragmentation patterns to other nodes, and the presence of smaller clusters with fewer than 10 nodes each. Conversely, at lower energies, a larger part of the nodes is grouped in big clusters, displaying almost three times less single nodes than at 70 eV. Moreover, at 25 eV, fewer clusters are present, but most of them contain many nodes (Figure 3).

Protonated beauvericin ions were consistently clustered together, regardless of the collision energy applied. However, as the collision energy increases (50 and 70 eV), the number of ions grouped within the protonated clusters increases. In contrast, sodiated ions are mostly found as singletons (self-loop nodes) or scattered in random groups at 25 eV. At increasing energies, beauvericin nodes are grouped into numerous clusters of only a few nodes. For instance, at 50 eV, three highly specific beauvericin clusters are formed and MS/MS pattern is similar to the protonated cluster at 25 eV, as it balances the presence of the fragments with the precursor ion. In all three clusters, the identification of both the conserved ions (*m/z* 266, 284, and 384) and the repetitive loss of amino acid residues at their peptide bonds (b-fragments) are equally important to determining similarity. At energies above 50 eV, nodes that contain conserved beauvericin ions are grouped into different clusters and the presence of these fragments are no longer exclusively what determines the similarity.

**FIGURE 4:**
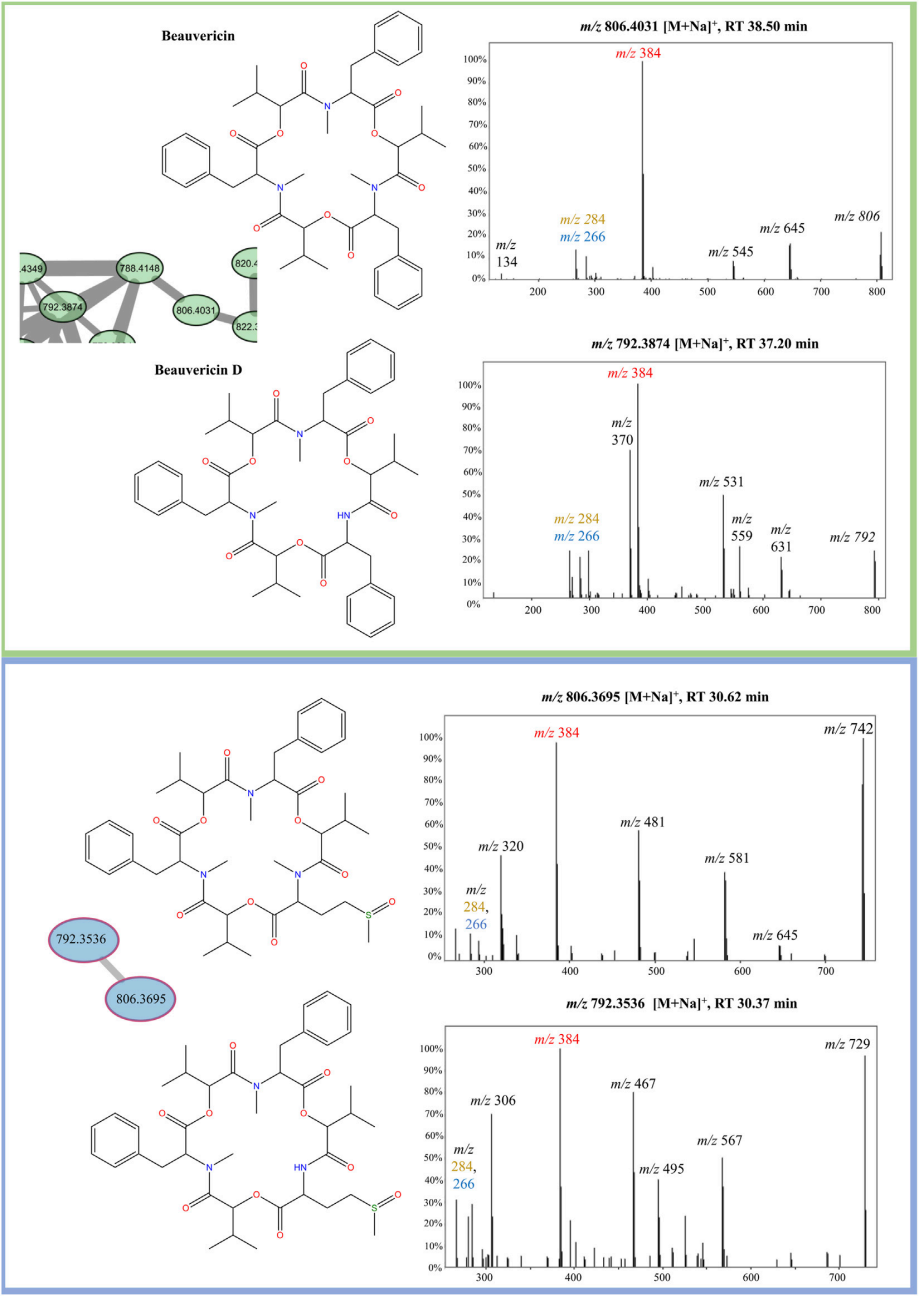
Elucidation of structural isomers from the beauvericin molecular class. Two sodiated ions at *m/z* 806 and *m/z* 792 have been clustered separately and contain different fragmentation patterns. The group colored in green belongs to the known compounds, beauvericin (*m/z* 806.4,031) and beauvericin D (*m/z* 792.3874) and elute at later retention time (∼38 min). The other group, colored in blue, elutes at earlier retention times (∼30 min) and belongs the to the novel beauvericin analogues (1, *m/z* 806.3695) and (2, *m/z* 792.3536). These metabolites contain an unusual methionine sulfoxide residue and a diagnostic loss of 64 Da, corresponding to the neutral loss of methanesulfenic acid. Both groups display the diagnostic beauvericin ions at *m/z* 266, 284, 384.

FBMN analysis of sodiated beauvericins at 70 eV revealed subclasses with distinct amino acid residues, allowing distinction between known beauvericins (beauvericin and beauvericin D) and two previously unknown structural isomers (Figure 4). Both clusters contain the conserved ions at *m/z* 266, 284, 384. However, the MS/MS spectra of the unknown compounds, eluting in earlier retention time (∼30 min), exhibit an additional neutral loss of 64 Da, which corresponds to the loss of methanesulfenic acid (CH_3_SOH) from the methionine sulfoxide residue (Supplementary Figure S1). Putative annotation of the first novel metabolite (compound 1) showed a sodiated mass of *m/z* 806.3695, repetitive losses of 161–100–161–100 (from the MePhe-Hiv-MePhe-Hiv residues) and an additional loss of 97 Da, which corresponds to the *N*-methylated methionine residue (MeMe(O)) after the loss of methanesulfenic acid. The proposed molecular formula is C_41_H_57_N_3_O_10_S, displaying a mass error below 3 ppm, and retention time of 30.62 min. Compound (2) had the same fragmentation pattern as compound 1 (losses of 161–100–161–100 Da), expect for the loss of 83 Da, correlated to the non-methylated methionine sulfoxide residue (Me(O)) after the loss of methanesulfenic acid. This new depsipeptide has a *m/z* of 792.3536 and proposed molecular formula C_40_H_55_N_3_O_10_S (mass error <3 ppm). Lastly, apart from the novel compounds, beauvericin D was also putatively annotated by MS/MS fragmentation. Although this molecule follows the exact same fragmentation pattern as beauvericin, its spectra also display a specific loss of 147 Da, which is characteristic of phenylalanine without any *N*-methylation. The putative MS/MS annotation of all four isomers are detailed in the Supplementary Materials (Supplementary Figures S2–5).

Overall, FBMN was successful in assisting the detection of beauvericin and other 13 analogues that share the same MS/MS fragmentation pattern. Among these molecules, some are known molecules that have been putatively identified, while others require further characterization and assessment of their unmatched fragment spectra. Table 1 shows the beauvericin ions identified for *F. oxysporum*, the proposed molecular formular and, when available, putative annotation.

**TABLE 1:** Beauvericin analogues identified by FBMN and MassQL in *Fusarium oxysporum* extracts. All ions have been clustered with known beauvericin analogues and contain at least one of the diagnostic ions for the beauvericin molecular class. Molecules have been annotated by comparison with curated databases. Compound 1 and 2 have never been reported in literature and its putative annotation is described in the Supplementary Materials. Ions with (*) were identified by MassQL only. Compounds that have not been putatively annotated had their molecular formula proposed.

	Exp. *m/z*	Theoretical *m/z*	Error (ppm)	RT (min)	Putative annotation	Proposed	Most abundant fragments at 50 eV
						Molecular formula	
1	792.352 [M + Na]^+^	792.3505	1.89	30.33	Compound 2	C_40_H_55_N_3_O_10_S	306, 384, 467, 567, 728, 792
2	806.3695 [M + Na]^+^	806.3662	4.09	30.61	Compound 1	C_41_H_57_N_3_O_10_S	320, 384, 481, 581, 742, 806
3	808.378 [M + Na]^+^	-	-	32.54	-	C_45_H_59_N_3_O_9_	284, 384, 547, 575, 647, 728, 808
4	822.396 [M + Na]^+^	822.3941	2.31	32.7077	Beauvericin J	C_45_H_57_N_3_O_10_	384, 400, 545, 563, 645, 663, 822
5	820.411 [M + Na]^+^	820.4149	4.75	33.74	Beauvericin A/F	C_46_H_59_N_3_O_9_	384, 398, 559, 659, 820
6	824.3755 [M + Na]^+^	-	-	35.03	-	C_45_H_59_N_3_O_10_	384, 402, 563, 663, 824
7	825.464 [M + Na]^+^	-	-	35.04	-	C_45_H_58_N_2_O_11_	402, 563, 663, 825
8	808.413 [M + Na]^+^	-	-	35.78	-	C_45_H_59_N_3_O_9_	206, 386, 547, 647, 808
9	788.432 [M + Na]^+^	-	-	36.24	-	C_48_H_58_N_3_O_7_	366, 384, 527, 627, 645, 788
10	822.3944 [M + Na]^+^	822.3941	0.36	36.39	Beauvericin J	C_45_H_57_N_3_O_10_	300, 384, 400, 563, 663, 822
11	778.3641 [M + Na]^+^	778.3679	4.88	36.85	Beauvericin G2	C_43_H_53_N_3_O_9_	356, 384, 517, 617, 778
12	758.3961 [M + Na]^+^	758.3992	4.08	37.10	Beauvericin E	C_41_H_57_N_3_O_9_	758, 597, 525, 497, 384, 336
13	792.387 [M + Na]^+^	792.3836	4.29	37.2159	Beauvericin D	C_44_H_55_N_3_O_9_	792, 631, 531, 384, 370
14	806.403 [M + Na]^+^	806.3992	4.71	38.17	Beauvericin	C_45_H_57_N_3_O_9_	806, 645, 545, 384
15	722.3968*	-	-	36.2046	-	C40H55N3O9	433, 362, 333, 300, 262, 134
16	750.4325* [M + H]^+^	750.4329	0.5	38.3355	Beauvericin K	C_42_H_59_N_3_O_9_	489, 362, 328, 262, 134
17	764.4355*	-	-	39.4984	-	C_43_H_61_N_3_O_9_	503, 603, 362, 342, 262, 134
18	780.4490*	-	-	34.5900	-	C_43_H_61_N_3_O_10_	537, 437, 262, 180

### 3.3 Finding beauvericin analogues with MassQL analysis

The MassQL tool was used to search for spectra containing specific fragments related to beauvericin. For this, MassQL used the FBMN with different CEs to search for a query in a mass spectrometry-centric fashion, targeting to find its related ions without ambiguity. Three different strategies have been applied to search for this molecular class and a summary with their description and their main findings are available on Table 2. The complete results generated from each query and the links for their jobs on GNPS are available on Supplementary Material and Supplementary Table S2.

**TABLE 2:** MassQL strategies used for the search of beauvericin analogues. Queries 1 and 2 search for product ion formation, whereas the third uses neutral loss or delta mass.

Query N	Description	Targeted IONS	Main findings
QUERY 1	Search for conserved ions individually	Protonated: *m/z* 362 or 262 or 244 or 134; Sodiated: *m/z* 384 or 284 or 266	112 (25 eV), 167 (50eV), and 156 (70 eV) scans
QUERY 2	Search for conserved ions systematically concatenated	Protonated: *m/z* 362 and 262 and 244; Sodiated: *m/z* 384 and 284 and 266	27 (25 eV), 54 (50eV), and 65 (70 eV) scans
QUERY 3	Search for a neutral loss sequence of MePhe → Hiv → MePhe residues	Addition of a delta mass of 161 (X+161), followed by 100 (X+261) and then 161 (X+422)	150 (25 eV), 212 (50eV), and 81 (70 eV) scans

The initial query (query 1) aimed to search for MS/MS spectra that incorporated the product ion mass of at least one of the conserved ions previously identified in the beauvericin analogues. Thus, the search was conducted using the protonated fragment ions c_2_, b_2_ and b_2_-H_2_O at *m/z* 362, 262 and 244, and their sodium adducts at *m/z* 384, 284 and 266, respectively. The y ion at *m/z* 134 was searched for protonated ions only. This query resulted in 112, 167, and 156 scans for CID energies of 25, 50, and 70 eV, respectively. Furthermore, some ions identified by this query did not exhibit the characteristic fragmentation pattern of the hexadepsipeptide class, exhibiting *m/z* values below 600 or above 900. For purpose of illustration, at 50 eV, one of the most abundant ion was at *m/z* 625, and its fragmentation produced the major product ions at *m/z* 449, 405, 378, 322, and 181, which are not in agreement with the fragmentation pattern of beauvericin analogs.

To enhance the specificity of the search for beauvericin analogues and to define a precise sequence of *N*-MePhe and *D*-Hiv residues, the second query was employed (query 2) where conserved ions were systematically concatenated. This methodology aimed to refine the search parameters, thereby providing a more targeted examination of potential analogues. Upon implementing this query, CID energy levels of 25, 50, and 70 eV yielded 27, 54, and 65 scans, respectively. The significant reduction in the number of returned MS/MS spectra points out the increased specificity of this search strategy.

Given the potential modifications at the N- or C-termini of beauvericin analogues, identifying all derivatives via pseudoprecursor ion scanning and specific product ion formation becomes challenging without priorly conducting a fragmentation study, as demonstrated previously. To address this challenge, an alternative method was employed (query 3), which involved searching for a neutral loss sequence that included at least one Hiv and two *N*-MePhe unit in the analogs. The MassQL query involved the addition of a delta mass of 161 (X+161), followed by 100 (X+261) and then 161 (X+422); this indicates the formation of a product ion at *m/z* = X, resulting from the sequential neutral loss of the MePhe→Hiv→MePhe residues. The query yielded 150, 212 and 81 scans for CID energies of 25, 50, and 70 eV, respectively. The higher number of identifications, compared to the results from the product ion scan (query 2), can be attributed to a greater sensitivity to mass deviation. This is because the query measures the relative mass loss between a precursor ion and a particular fragment ion. For example, in the dataset obtained at 50 eV, the query successfully pinpointed the beauvericin derivatives with precursor ions at *m/z* 784 [M + H]^+^ (RT = 30.59 min), and *m/z* 784 [M + H]^+^ (RT = 38.19 min), returning 12 scans and 2 scans for each respective MS/MS spectrum.

Given that the most promising results were obtained from the second and third queries, and to simplify the comparison of query results while avoiding redundancy, molecular networks were constructed using data derived from MassQL (Supplementary Figure S6). The dataset from each CID energy (25, 50, and 70 eV) contributed to the formation of distinctive clusters within the molecular network, displaying similar results as the preliminary FBMN. Upon visual inspection of the precursor ion clusters generated based on the proposed queries, it was observed that sodiated ions demonstrated improved clustering at higher fragmentation energies, with the most optimal clustering seen at a CID energy of 70 eV. On the contrary, protonated ions presented an opposite pattern. These findings are consistent with the FBMN results previously presented. In the context of sodiated ions clustering, query 03 appeared to be more effective, whereas query 02 identified two precursor ions at *m/z* 676 and 748 that do not fall within the beauvericin analogs. Furthermore, the identified sodiated precursor ions align with those manually analyzed in the molecular network generated by the FBMN approach.

With regard to protonated ions, query 02 demonstrated superior selectivity. Analyzing the molecular networks of the protonated ions at collision energies of 25 and 50 eV, it was possible to identify precursor ions that were not distinguished at the higher energy level or for the corresponding sodiated ions. The analysis of MS/MS mass spectra for the precursor ions identified in these clusters, specifically at *m/z* 722, m*/z* 750, m*/z* 764, and *m/z* 780, showed fragmentation patterns characteristic of beauvericin analogues (Supplementary Figure S7; Table 1). These precursor ions demonstrated similar fragmentations, due to the formation of the product ions at *m/z* 362, 262, and 134, along with consecutive neutral losses of 161 Da and 100 Da. As an illustration, the precursor ion at *m/z* 750 generated sequential neutral losses of 161, 100, and 127 Da; the neutral loss of 127 Da suggests that this ion contains an N-leucine/isoleucine unit in place of a phenylalanine unit (Xu et al., 2016). The annotation derived from structure-based propagation for the other ions implies that the modification occurs at the amino acid unit, as indicated by the observed mass differences of 128, 141 and 157 Da.

## 4 Discussion

### 4.1 Finding conserved ions by PCA-based molecular fingerprinting

Initially, we conducted PCA analysis on the MS/MS data, revealing a positive and correlation between collision energy and the percentage of variance explaining the presence of beauvericin. This correlation can be attributed to the promotion of successive cleavages at increasing energies, leading to the generation of building block structures (‘ions”) formed by single amino acid residues. This evidence is more prominent by analysis of PC1, which contains the highest explained variance of all PCs, embracing 21.22%, 26.70%, and 73.69% of the whole abundance variability.

PCA analysis for high collision energies data showed that beauvericin analogues were grouped based on the type of adduct (protonated or sodiated). Based on this distinction, two sets of particular fragments were discovery for the identification of these hexadepsipeptides: ([M + H]^+^) *m/z* 134, 244, 262, and 362 and ([M + Na]^+^) *m/z* 266, 284 and 384. To date, numerous LC-MS/MS methods have been developed to detect enniantins and beauvericins. However, most of these methods target the specific congeners at *m/z* 362, or semi-targeted relying on one or two product ions. Consequently, identifying a total of seven diagnostic fragments could assist in the identification of this molecular class, enabling a more automatic detection of analogues, reducing analysis time and minimizing compound overestimation.

Interestingly, sodiated adducts exhibited a lower overall abundance of the fragments, implying that these adducts are less prone to fragmentation than their protonated counterparts. This limited number of fragments can be attributed to the chelation effect of the carbonyl groups from the six amino acid residues with the sodium ion, further stabilizing the cycle and increasing the energy necessary for the cleavage of the peptides. On the one hand, this chelation effect makes the annotation of known beauvericin more straightforward given the spectra usually contains only b2-fragments, which preserve the entire amino acid residue. However, for unknown analogues, the information derived by other fragments in the protonated MS/MS spectra can aid in the determination of the right amino acid residues based on specific losses. The MePhe residue (*m/z* 134) was the only ion that appeared in both sodiated and protonated forms. This fragment thus represents the sole conserved ion presents in both adducts, making it a valuable fragment for this molecular class.

While this unsupervised analysis provided valuable insight into the presence of class-conserved ions, PCA alone falls short of clustering molecular features based on their fragmentation patterns, thus failing to identify compounds with similar MS/MS losses. Furthermore, the number of observed protonated and sodiated ions in the PCA is lower than the actual number of beauvericin analogues, and conserved ions often go unnoticed in lower intensity spectra. This limitation presents challenges, particularly for molecular classes that lack class-specific fragments. In such cases, a comprehensive assessment of the MS/MS data requires the integration of complementary tools that incorporate similarity outputs into the analysis process.

### 4.2 Exploration of beauvericin analogues by FBMN

Molecular networking has been widely used in natural product research to visualize and interpret untargeted MS/MS data. This algorithm is based on principle that structurally related molecules tend to exhibit similar fragment patterns. As a result, MS/MS spectra can be mapped as molecular networks by comparing the spectral similarity between each pair of spectra. Given the structural relation of beauvericin analogues, these cyclic depsipeptides generate highly similar fragment patterns in tandem mass spectrometry. Consequently, these patterns can be organized into molecular families within the network, facilitation the annotation of known novel analogues. This approach has already been successfully applied to beauvericin and enniatin analogues, leading to the discovery of four enniatins and three beauvericin-producing fungi, as well as the identification of one new isomers of enniatin A and three new bassianolide analogs (Li et al., 2020).

Although MN has been extensively employed for compound mapping, only a few studies have used this approach to investigate fragmentation differences within a specific molecular class. Varying collision energies for the same molecule renders distinct MS/MS spectra, providing a deeper understanding of the chemical structure, fragment stability, and the accurate compound characterization.

To address the fragmentation pattern variations of beauvericin under different collision energies, we subjected each dataset (25, 50, and 70 eV) to feature-based molecular networking. This approach provided a visual representation of the clustering pattern and revealed differences between subclasses and adducts. For instance, the lower number of singletons at lower collision energies illustrates the reduced fragmentation at 25 eV, resulting in spectra formed mainly by the precursor ions and a weaker contribution of the fragments for overall fragmentation pattern. Moreover, at higher CID energies, there is a higher number of clusters that contain less than 10 nodes. This indicates that the increased number of fragments enhances specificity of the MS/MS spectra, differentiating fragmentation patterns even within the same molecular class.

Interesting, when we conducted a targeted search of the ions clustered on the PCA analysis for sodiated and protonated adducts, we observed a similar separation within MN. This finding was unexpected, considering that both types of ions theoretically have the same tendency of losing amino acid residues at the peptide bond. However, this discrepancy highlights the capacity of FBMN to distinguish classes not only based on their primary losses (such as b-fragments), but also on the more subtle spectral differences, as the presence of a and c-fragments, conserved ions, and losses specific to particular amino acid residues.

**FIGURE 5:**
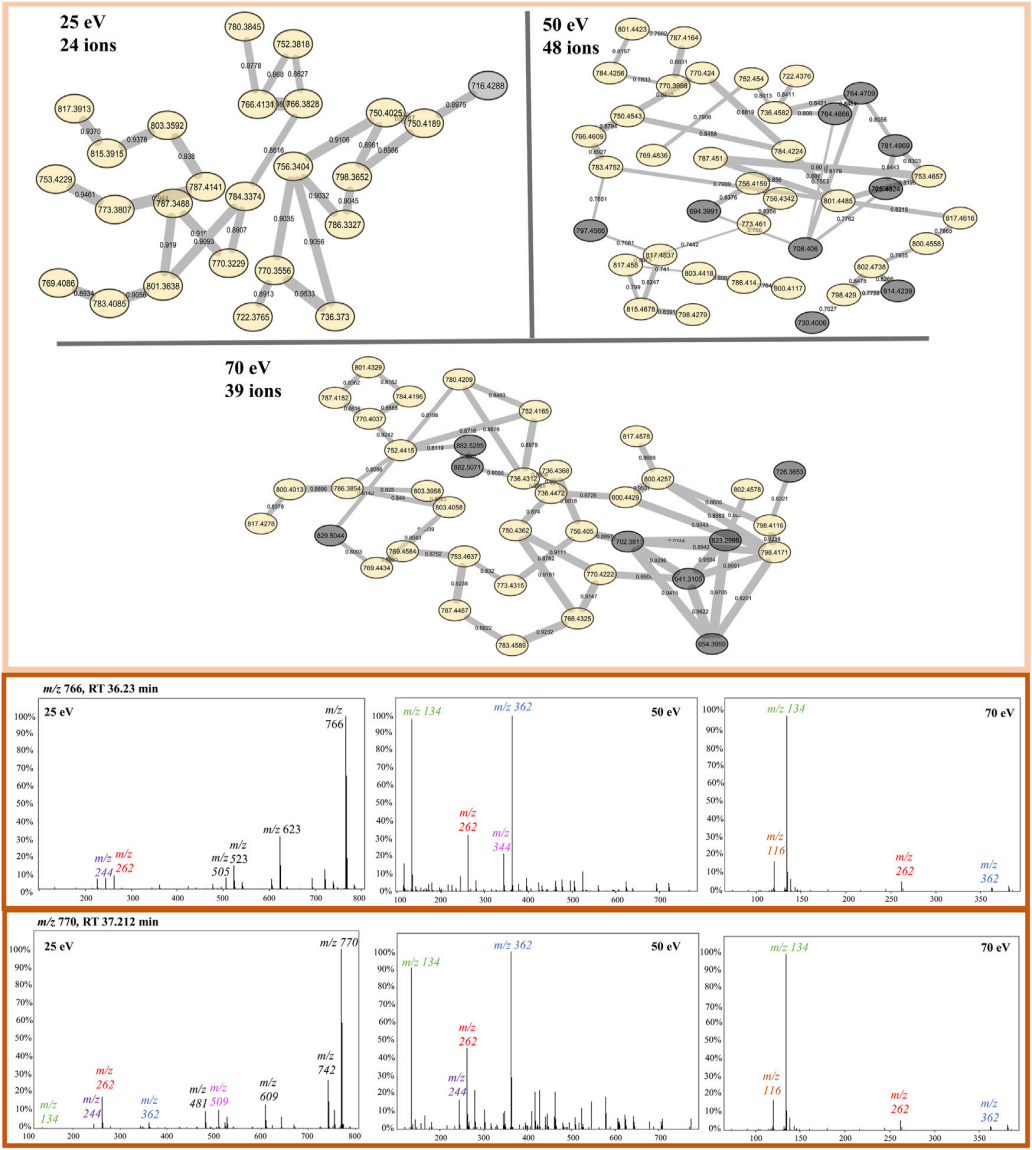
Protonated beauvericin clusters from the FBMN at 25, 50, and 70 eV. Nodes colored in yellow are found in all CIDs whereas nodes colored in gray are specific for only one CID. Example of the most abundant ions found on the protonated cluster. MS/MS spectra of the ions *m/z* 766 and *m/z* 770 are sequentially shown in all three collision energies. The diagnostic fragments for this molecular class are colored in the spectra as *m/z* 134, 244, 262, and 362.

The protonated ions of beauvericin were consistently clustered together, irrespective of the collision energy applied (Figure 5). This suggests that the beauvericin analogues exhibit both diagnostic fragments and similar fragmentation patterns even at lower CID energies. However, a closer examination of the protonated cluster revealed that the highest number of ions were obtained at 50 eV. This improved accuracy in identifying this molecular class can be attributed to presence of MePhe, MePhe-Hiv, and Hiv-MePhe-Hiv fragment peaks at similar abundances (ranging from 50%–100%). At 70 eV, the cleavage of fragments containing two and three amino acid residues, increases the abundance and prevalence of the single-residue MePhe fragment. Consequently, other peptides that do not belong to the beauvericin analogs but also contain MePhe residues, such as pentacyclic peptides at *m/z* 522, 541, and 654, are clustered in the same group. This increases the specificity of peptides as opposed to specifically finding beauvericin hexadepsipeptides. Lastly, despite the protonated clusters at 25 eV contains the lowest number of ions grouped together, this ionization energy gives the most detailed chemical information about the amino acid residues, displaying the majority of the b-fragments and thereby facilitating the annotation of known and novel compounds (Figure 5).

**FIGURE 6:**
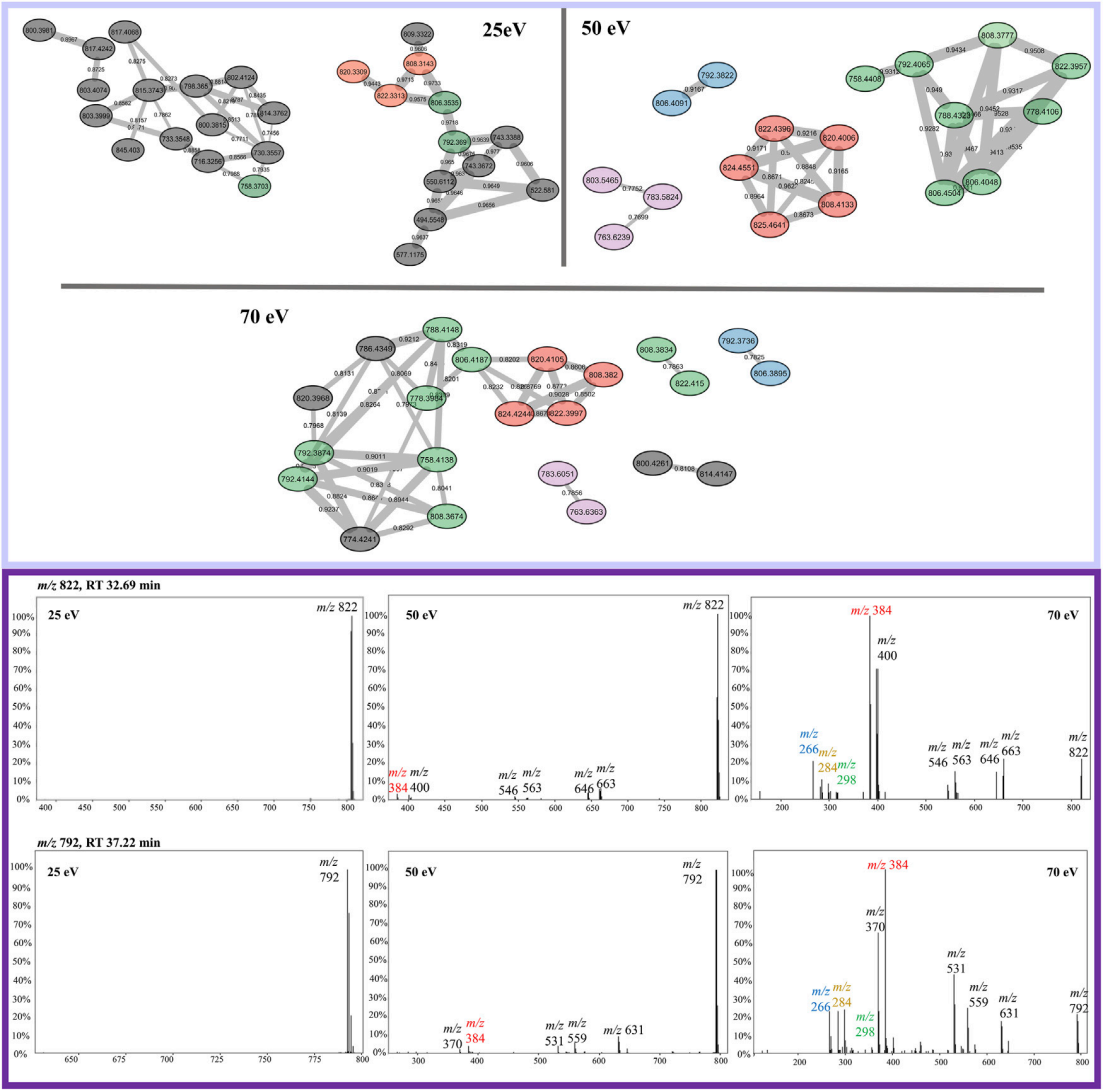
Sodiated beauvericin clusters from FBMN at 25, 50, and 70 eV. Nodes can be tracked by their color in all three collision energies, except for the nodes colored in gray which are specific for only one CID energy. Example of the most abundant ions found on the protonated cluster. MS/MS spectra of the ions *m/z* 822 and *m/z* 792 are sequentially shown in all three collision energies. The diagnostic fragments for this molecular class are colored in the spectra as *m/z* 266, 284, and 384.

The opposite scenario is encountered for sodiated beauvericin ions. At low CID energies, the fragmentation of these molecules is insufficient due to the chelation effect on the sodium ion. Consequently, beauvericin nodes either appear as singletons or clustered with nodes from other molecular classes that also do not fragment. At higher energies, MS/MS spectra contains a higher number of fragments. Therefore, its FBMN reveals the clustering of analogs based on their diagnostic fragmentation, resulting in smaller and more specific clusters (Figure 6). At 50 eV, the MS/MS spectra exhibited all the conserved ions (*m/z* 266, 284, and 384), leading to the most accurate detection of analogs. At energies above 50 eV, the specificity of these conserved ions diminishes in favor of providing information about amino acid residues other than MePhe and Hiv. This results in the formation of a larger number of small networks representing subclasses of beauvericin, which encompass the ions at *m/z* 266, 284, and 384 as well as residue-specific fragmentations.

The analysis of sodiated ions at high collision energies allowed the identification of two clusters that contain precursor masses of *m/z* 792 and 806. The first was annotated as beauvericin and its analogue beauvericin D. The latter has been previously isolated from *Beauveria* species (Fukuda et al., 2004), however, it has never been reported for *F. oxysporum*. The second group had their metabolites elucidated as the novel beauvericin analogues (MePhe-Hiv-MePhe-Hiv-MeMet(O)-Hiv and MePhe-Hiv-MePhe-Hiv-Met(O)) that contain an unusual methionine sulfoxide residue. Both compounds have never been reported for this species, although Gunasekera and co-workers have already shown the potential of bacteria to produce cyclic depsipeptides with this type of residue (Gunasekera et al., 2008). Putative annotation was mainly based on the neutral loss of 64 Da, which has been previously associated to the loss of methanesulfenic acid from precursors containing the oxidized methionine Met(O) residue (Pilo and McLuckey, 2014). This neutral loss is unique to Met(O) and was essential to differentiate between this residue and phenylalanine, given that both contain the same nominal mass.

Lastly, FBMN enabled the identification of beauvericin and other 13 analogues that share the same MS/MS fragmentation pattern. These include known analogues that have not been described for this species and unknown molecules, highlighting the potential of the molecular networking for the identification of novel secondary metabolites, as well as F. oxysporum potential to produce hexadepsipeptides.

Overall, FBMN provided valuable insights in several areas: (1) establishing the relationship between adducts and the fragmentation patterns, (2) identifying clusters specific to beauvericin, (3) characterizing both known and unknown analogues, and (4) differentiating between structural isomers. However, the extensive data generated by LC-MS/MS analysis, along with the occurrence of multiple fragmentation events during mass spectrometry analysis, can complicate the molecular networking process leading to inaccuracies in clustering and annotations. The fragmentation patterns, depending on the collision energy, may result in clustering of metabolites that are not exclusive to the beauvericin family, as observed for the sodiated ions at 25 eV (where no fragments were formed) and the protonated cluster at 70 eV (where only one abundant fragment was formed). Moreover, without prior knowledge derived from PCA regarding diagnostic fragments, determining the optimal collision energy for the FBMN and identifying its clusters become limiting, relying solely on manual validation and visual inspection of the MS/MS spectra.

### 4.3 Identification of beauvericin analogues by MassQL

Based on the analysis of the fragmentation pattern of beauvericin analogues and the insights derived from PCA and FBMN analysis, a series of investigations were conducted using the MassQL tool. The goal of this investigative process was to demonstrate the functionality of MassQL and to highlight its significant potential for mining beauvericin-like structures. The implementation of this tool was specifically designed to automate the search for spectra containing specific fragments associated with beauvericin. The automation facilitated by MassQL streamlines the identification process, enhancing the efficiency and accuracy of beauvericin analogue detection.

The application of combined multiple ion search proved to be a potent strategy to refine the search parameters, thereby increasing specificity and selectivity in the search for beauvericin analogues. Conserved ions serve as distinctive signatures in mass spectrometry studies, which enable efficient identification and grouping of compound analogues. This principle of structural similarity allows for a more targeted and efficient search within complex datasets, reducing false positives and enhancing the discovery of known and potentially novel analogues. However, the effectiveness of this method relies heavily on a thorough understanding of the fragmentation behavior of the compound class, and accurate identification of the conserved ions. Therefore, careful interpretation and validation of results are key to ensuring the correct grouping of analogues and the discovery of potential new ones.

The neutral loss process, on the other hand, focused on identifying specific mass differences between the precursor ion and its subsequent fragment ions, corresponding to the loss of a particular neutral moiety during fragmentation. This approach can thus account for variability in fragmentation patterns. If a precursor ion can lose a specific group or moiety during fragmentation, the resulting mass difference can be tracked across various spectra, regardless of the exact identity of the fragment ions.

Neutral loss searches allowed for the identification of a broader range of compounds that contain the same functional group or moiety, even if their overall structures are different. This is especially important when studying a family of compounds, like beauvericin analogues, where subtle structural differences can lead to significant variations in fragmentation pattern.

These different strategies demonstrate the adaptability of mass spectrometry-based techniques for compound identification. Using an ion-focused approach to conserved products can provide high selectivity, which is beneficial when studying specific subclasses or known structures. On the other hand, a strategy focused on neutral losses can be employed when the goal is a more exhaustive search in a class of compounds. It is the careful choice and combination of these strategies that allows for a robust and differentiated exploration of complex mass spectrometry datasets.

## 5 Conclusion

In the present study, we demonstrate the potential of combining PCA, spectral similarity networking and MassQL as an effective approach for decoding mass fragmentation pathways of beauvericins. To achieve this objective, we employed these approaches in a combination of different collision energies from ESI-MS/MS experiments from a *F. oxysporum* extract to determine both the mass fragmentation pathways and the identification of known and novel hexadepsipeptides.

PCA and FBMN offered great insights into the correlation of the type of ions (protonated and sodiated adducts) and fragmentation patterns. Sodiated ions have a chelation effect on the sodium and the carbonyl from the peptide bond, further stabilizing the cycle and increasing the energy necessary for fragmentation. Based on this distinction, two sets of particular fragments were discovery for the identification of these hexadepsipeptides: ([M + H]^+^) *m/z* 134, 244, 262, and 362 and ([M + Na]^+^) *m/z* 266, 284 and 384. Currently, most methods for the screening of beauvericin are targeted to a few product ions. Hence, the identification of these seven diagnostic fragments could help in the search for this molecular class, decreasing analysis time and overestimation of compounds.

By using these fragments, MassQL accurately found other analogues of the same molecular class, identifying 18 beauvericins in this fungi extract, including 4 which were not found when analyzing FBMN alone. The superior potential of MassQL in detecting beavuericin analoges is due to the direct search of diagnostic ions, overcoming the inaccuracies in clustering and annotations of FBMN caused by the occurrence of multiple fragmentation events during MS analysis. Hence, the implementation of this tool enabled the automated search for beauvericins, streamlining the identification process and enhancing the efficiency and accuracy of analogue detection.

Lastly, FBMN analysis of sodiated beauvericins at 70 eV revealed subclasses with distinct amino acid residues, allowing distinction between beauvericins (beauvericin and beauvericin D) and two previously unknown structural isomers with an unusual methionine sulfoxide residue (MePhe-Hiv-MePhe-Hiv-MeMe(O)-Hiv and MePhe-Hiv-MePhe-Hiv-Me(O)-Hiv). Beauvericin D has been previously isolated from *Beauveria* species; however, it has never been reported for *F. oxysporum*. Similarly, these novel compounds had never been reported for fungi species, although previous studies have already shown the potential of bacteria to incorporate these residues into cyclic depsipeptides.

Ultimately, this approach revealed the correlation between adducts and the fragmentation patterns, the identification of beauvericin clusters, the characterization of known and unknown analogs, and the differentiation between structural isomers. The combination of different tools could shed light on conserved MS characteristics, facilitating the identification of metabolites in complex mixtures.

## Data Availability

The datasets presented in this study can be found in online repositories. The names of the repository/repositories and accession number(s) can be found below: https://massive.ucsd.edu/ProteoSAFe/dataset.jsp?accession&equals;MSV000091616, MSV000091616.
