# Supplementary material for: Application of feature-based molecular networking and MassQL for the MS/MS fragmentation study of depsipeptides
Source: Front Mol Biosci. 2023 Aug 1;10:1238475. doi: 10.3389/fmolb.2023.1238475 (PMC10427501; doi:10.3389/fmolb.2023.1238475)
Supplement: Supplementary file 1 [file Table1.DOCX]

Supplementary Material

Application of Feature-based Molecular Networking for the MS/MS fragmentation study of depsipeptides

Denise M. Selegato*, Ana C. Zanatta, Alan César Pilon, Juvenal H. Veloso, Ian Castro-Gamboa

*** Correspondence:** Denise Medeiros Selegato: denise.selegato@embl.de

# Supplementary Data

**SIRIUS4 Command for metabolite annotation**

config --IsotopeSettings.filter true --FormulaSearchDB --Timeout.secondsPerTree 0 --FormulaSettings.enforced HCNOP --Timeout.secondsPerInstance 0 --AdductSettings.detectable [[M + H3N + H]+, [M + Na]+, [M - H2O + H]+, [M + H]+, [M - H4O2 + H]+] --UseHeuristic.mzToUseHeuristicOnly 650 --AlgorithmProfile qtof --IsotopeMs2Settings IGNORE --MS2MassDeviation.allowedMassDeviation 10.0ppm --NumberOfCandidatesPerIon 1 --UseHeuristic.mzToUseHeuristic 300 --FormulaSettings.detectable B,Cl,Br,Se,S --NumberOfCandidates 10 --AdductSettings.enforced , --AdductSettings.fallback [[M + Na]+, [M - H2O + H]+, [M + H]+, [M - H + Na + Na]+] --FormulaResultThreshold true --InjectElGordoCompounds false --StructureSearchDB BIO,METACYC,CHEBI,COCONUT,ECOCYCMINE,GNPS,HMDB,HSDB,KEGG,KEGGMINE,KNAPSACK,MACONDA,MESH,NORMAN,UNDP,PLANTCYC,PUBCHEM,PUBMED,YMDB,YMDBMINE,ZINCBIO --RecomputeResults false formula fingerprint structure.

**Selection of tolerance used in FBMN and MassQL**

The tolerance used for feature-based Molecular Networking and MassQL were based on non-strict tolerance levels recommended for QToF instruments. Moreover, the precursor ion mass tolerance is set at lower tolerance than MS/MS because beauvericin identifications were improved when spectra were searched with a wide mass tolerance window and precursor mass is used as a filter to discard incorrect matches.

**MassQL Queries**

| **Query 01** |
| --- |
| **MassQL Query** |
| QUERY scaninfo(MS2DATA) WHERE MS2PROD=(362.2 OR 262.1 OR 244.1 OR 134.1):TOLERANCEMZ=0.1  \|\|\|  QUERY scaninfo(MS2DATA) WHERE MS2PROD=(384.2 OR 284.1 OR 266.1):TOLERANCEMZ=0.1 |
| **Query Parse Visualization** |
|  |
| **Query 02** |
| **MassQL Query** |
| QUERY scaninfo(MS2DATA) WHERE MS2PROD=362.2:TOLERANCEMZ=0.1 AND MS2PROD=262.1:TOLERANCEMZ=0.1 AND MS2PROD=262.1 - formula(H2O):TOLERANCEMZ=0.1 AND MS2PROD=134.1:TOLERANCEMZ=0.1  \|\|\|  QUERY scaninfo(MS2DATA) WHERE MS2PROD=384.2:TOLERANCEMZ=0.1 AND MS2PROD=284.1:TOLERANCEMZ=0.1 AND MS2PROD=284.1 - formula(H2O):TOLERANCEMZ=0.1 |
| **Query Parse Visualization** |
|  |

| **Query 03** |
| --- |
| **MassQL Query** |
| QUERY scaninfo(MS2DATA) WHERE  MS2PROD=X AND MS2PROD=X+100.1:TOLERANCEMZ=0.1:INTENSITYPERCENT=1 AND MS2PROD=X+261.1:TOLERANCEMZ=0.1:INTENSITYPERCENT=1 |
| **Query Parse Visualization** |
|  |

# Supplementary Figures and Tables

## Supplementary Figures

**(A)**


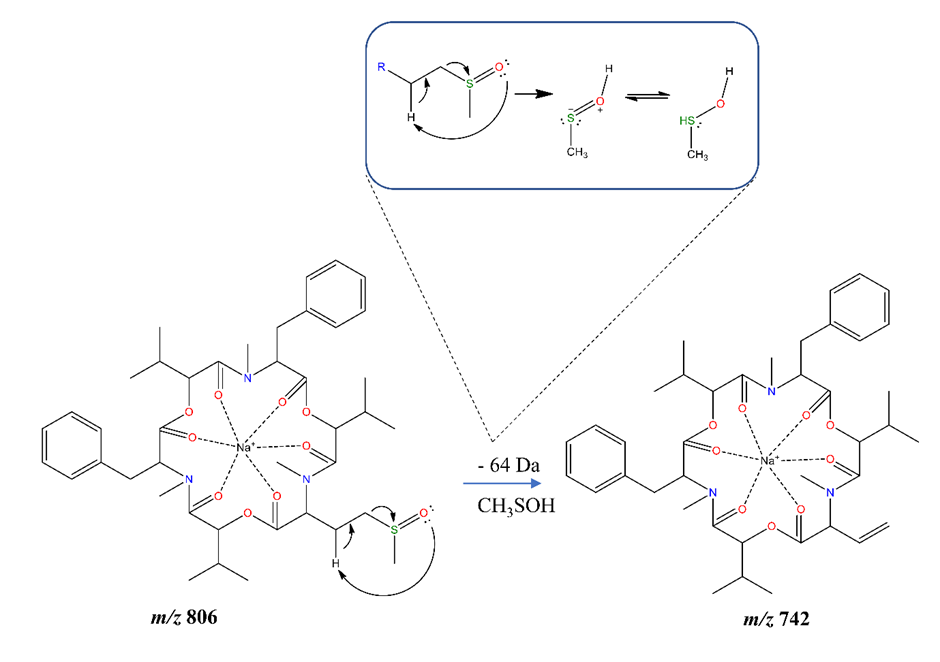

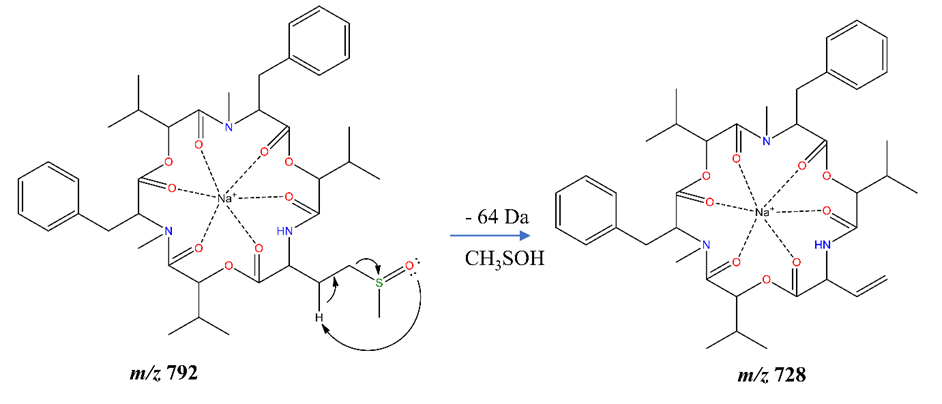


**(B)**


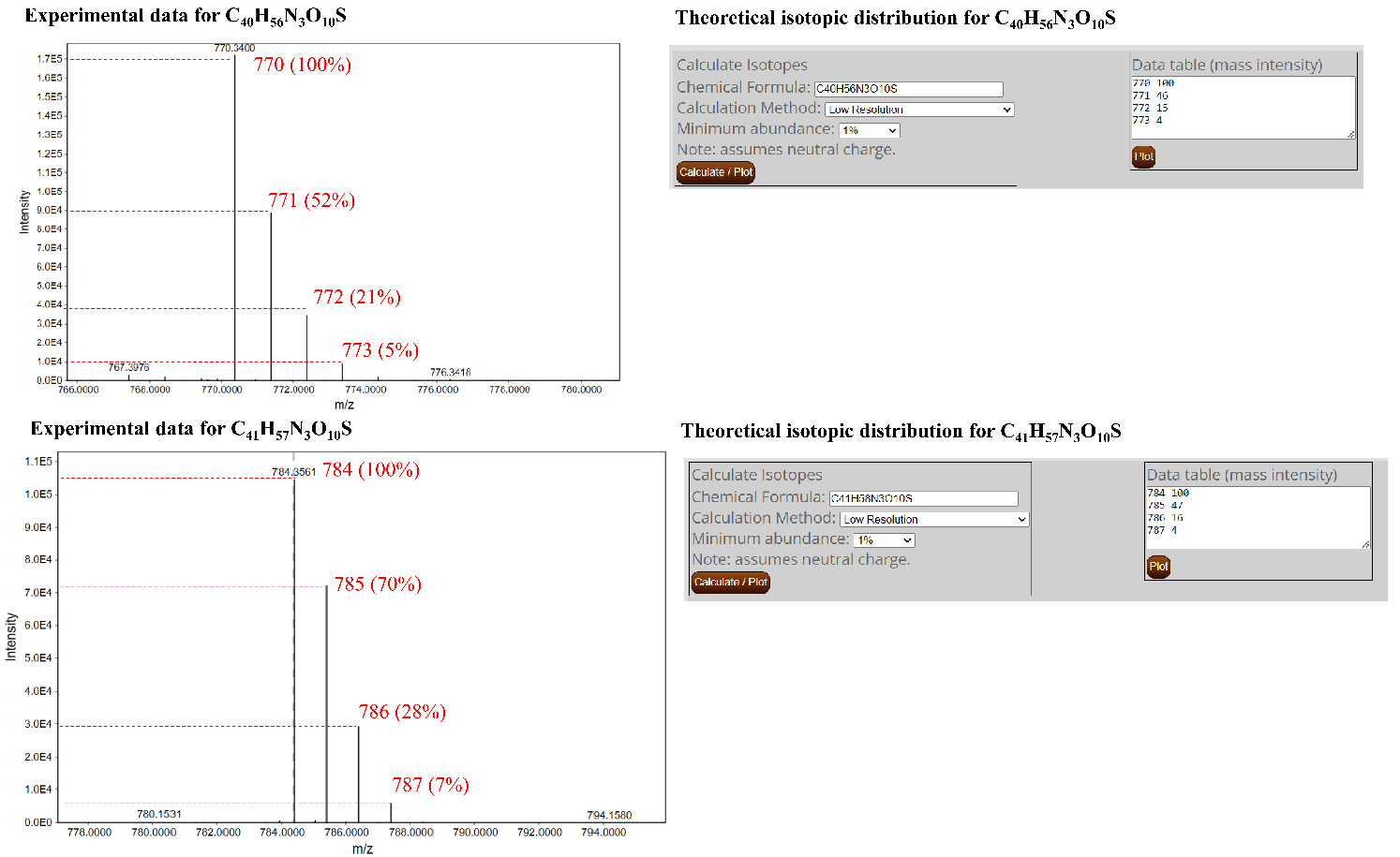


**Supplementary Figure 1.** (A) The rearrangement mechanism for the neutral loss of 64 Da in the methionine sulfoxide residue. (B) Comparison between theoretical and experimental isotopic distribution of compounds 1 and 2.


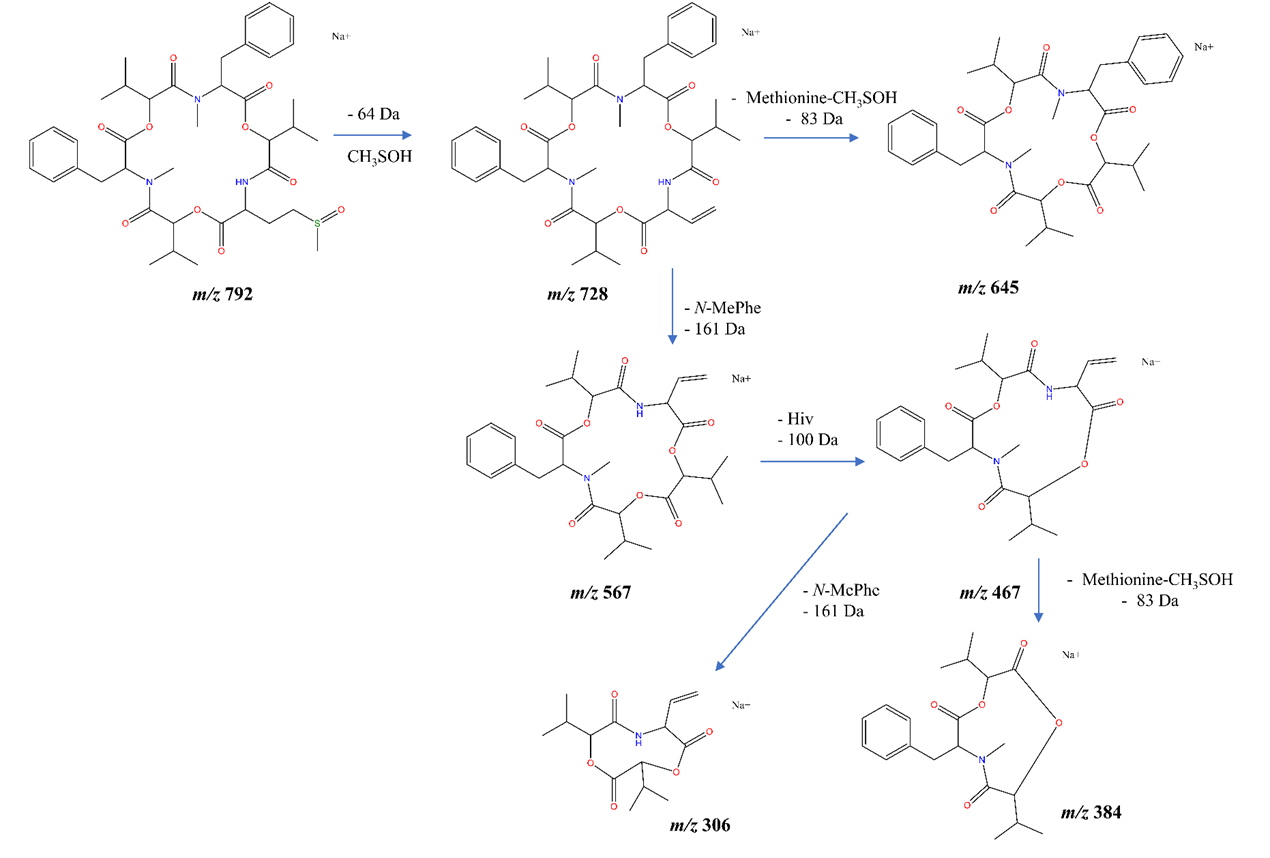


**Supplementary Figure 2.** ESI-MS/MS fragmentation scheme for *m/z* 792.3536 [M+Na]^+^, RT 30.37 minutes. Proposed molecular formula: C_40_H_55_N_3_O_10_S (mass error of 3 ppm).


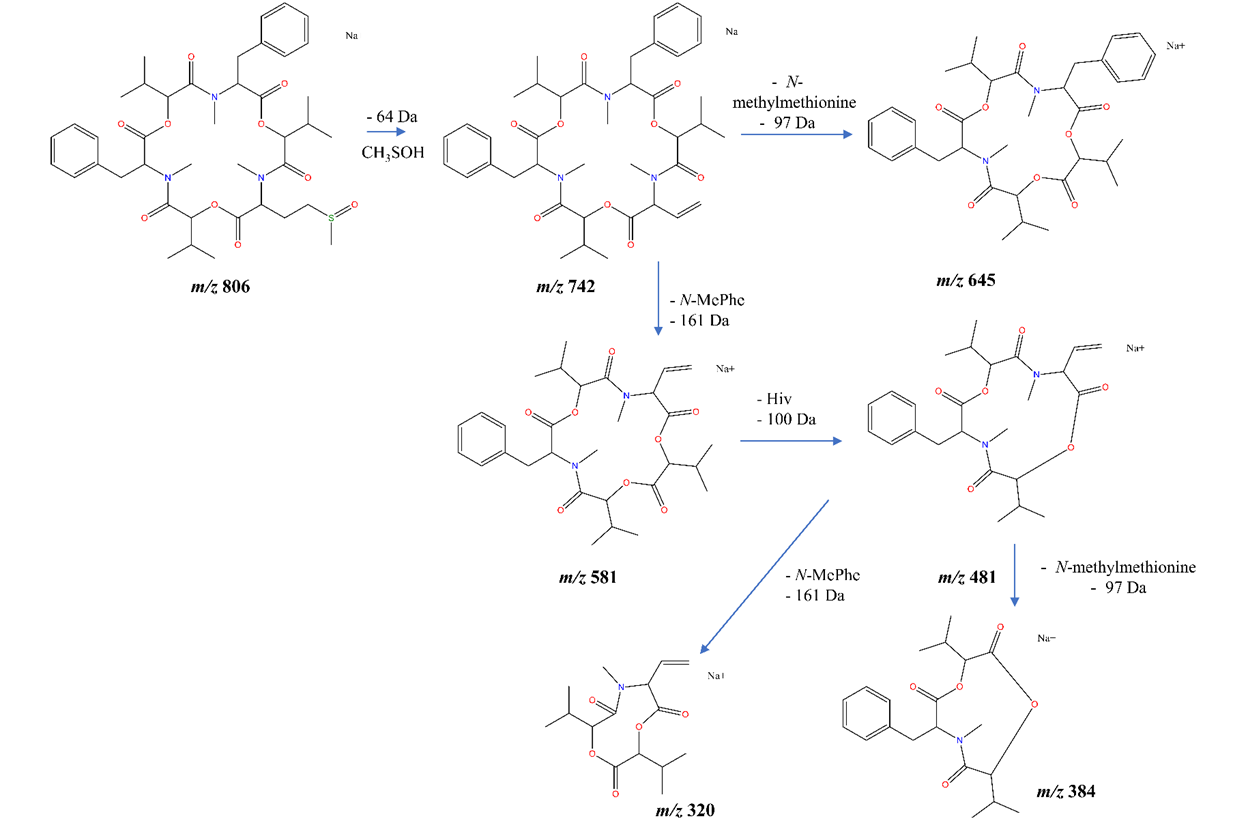


**Supplementary Figure 3.** ESI-MS/MS Fragmentation scheme for *m/z* 806.3695 [M+Na]^+^, RT 30.62 minutes. Proposed molecular formula: C_41_H_57_N_3_O_10_S (mass error of 3 ppm).


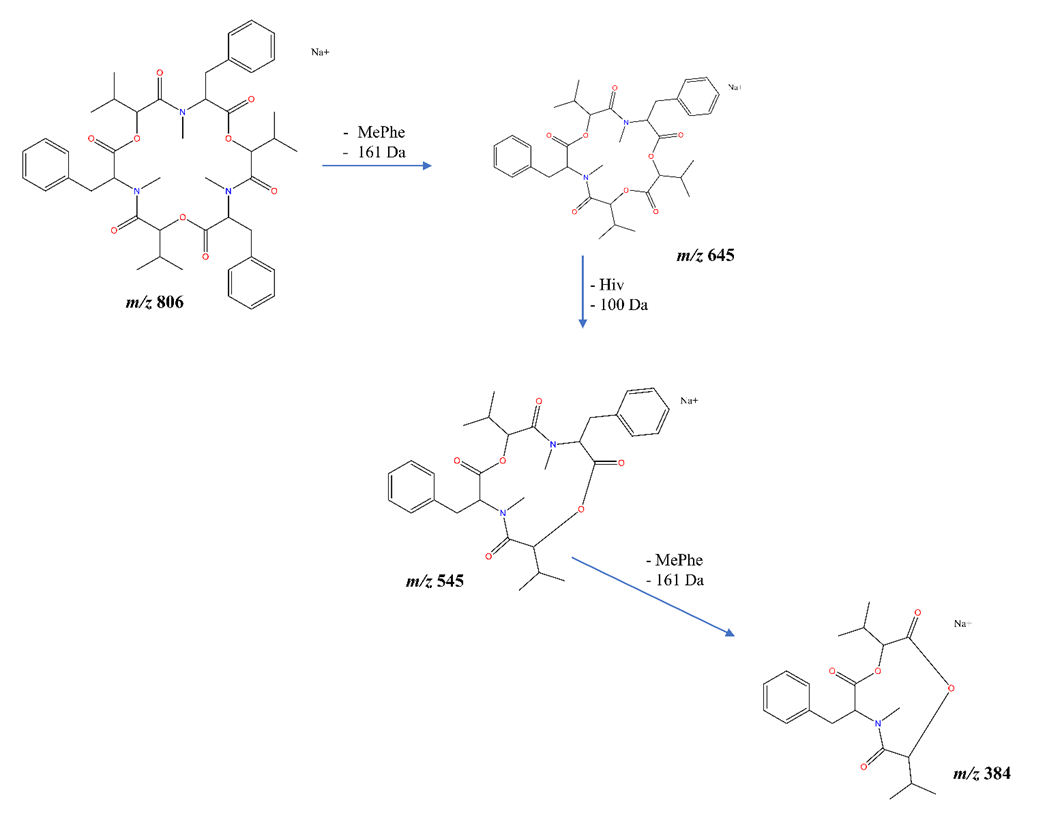


**Supplementary Figure 4.** ESI-MS/MS Fragmentation scheme for *m/z* 806.4031 [M+Na]^+^, RT 38.50 minutes. Proposed molecular formula: C_45_H_57_N_3_O_9_ (mass error of 4.83 ppm).


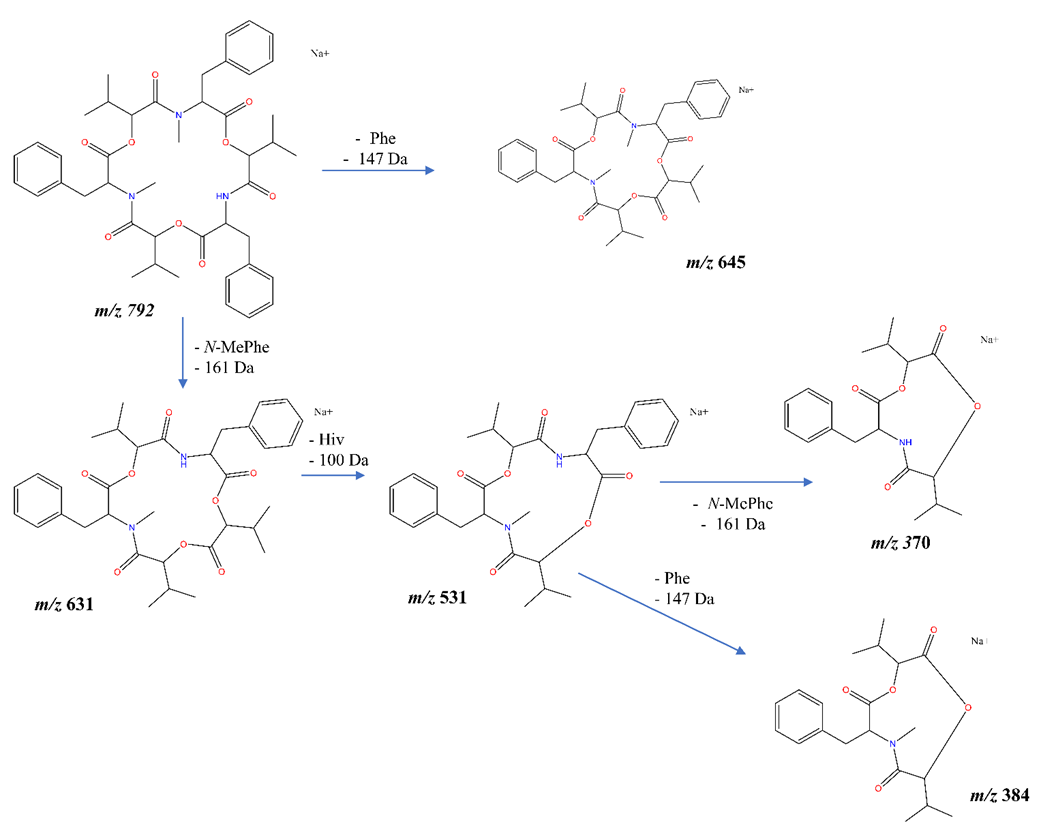


**Supplementary Figure 5.** ESI-MS/MS Fragmentation scheme for *m/z* 792.3874 [M+Na]^+^, RT 37.2 minutes. Proposed molecular formula: C_44_H_55_N_3_O_9_ (mass error of 4.79 ppm).

|  | **25 eV** | **50 eV** | **70 eV** |
| --- | --- | --- | --- |
| **A** |  |  |  |
| **B** |  |  |  |

**Supplementary Figure 6.** MassQL-FBMN clusters at 25, 50, and 70 eV for **(A)** query 2 and **(B)** query 3. Protonated precursor ions are colored in yellow and sodiated ions colored in red.

**A**

**B**

**Supplementary Figure 7.** **(A)** Protonated beauvericin cluster from the MassQL-FBMN at 50 eV. Nodes colored in grey are found in all CIDs whereas nodes colored in yellow are specific for only protonated clusters. **(B)** MS/MS spectra of the precursor ions at *m/z* 722, *m/z* 750, *m/z* 764, and *m/z* 780 at collision energy of 50 eV.

## Supplementary Tables

**Supplementary Table 1.** Data availability.

| **Type of Data** | **Platform** | **Link** |
| --- | --- | --- |
| Raw Data | **MassIVE** | https://massive.ucsd.edu/ProteoSAFe/dataset.jsp?accession=MSV000091616  ftp://massive.ucsd.edu/MSV000091616 |
| LC-MS/MS data processing and batch | **MassIVE** | https://massive.ucsd.edu/ProteoSAFe/dataset.jsp?accession=MSV000091616  ftp://massive.ucsd.edu/MSV000091616 |
| FBMN 70 eV | **GNPS** | https://gnps.ucsd.edu/ProteoSAFe/status.jsp?task=28563ce9301b47ed8e042548567fb38f |
| FBMN 50 eV | **GNPS** | https://gnps.ucsd.edu/ProteoSAFe/status.jsp?task=941df39105424f95a5bd0efbe2549315 |
| FBMN 25 eV | **GNPS** | https://gnps.ucsd.edu/ProteoSAFe/status.jsp?task=9e914444b87b44d79c9b7e00b949a7bb |

**Supplementary Table 2.** MassQL queries using product ion formation (Queries 1 and 2) and neutral losses (Query 3).

| **Query** | **Collision**  **energy** | **Query Link** | **nº of scans detected** | **FBMN link** | **Networking statistics** |
| --- | --- | --- | --- | --- | --- |
| **01**  **Product ion**  **formation**  (*m/z* 362 OR 262 OR 244 OR 134\|  *m/z* 384 OR 284 OR 266) | 25 eV | https://proteomics2.ucsd.edu/ProteoSAFe/status.jsp?task=1b07b2246fc540f69a41ed2844645ed3 | 112 | - | - |
|  | 50 eV | https://proteomics2.ucsd.edu/ProteoSAFe/status.jsp?task=a8a3cc24289841a38db7e18fa8d44b70 | 167 | - | - |
|  | 70 eV | https://proteomics2.ucsd.edu/ProteoSAFe/status.jsp?task=0d0235a8c276424dbf10c5ab40a3dbcc | 156 | - | - |
| **02**  **Product ion**  **formation**  (*m/z* 362 AND 262 AND 244 AND 134\|  *m/z* 384 AND 284 AND 266) | 25 eV | https://proteomics2.ucsd.edu/ProteoSAFe/status.jsp?task=81b601d243284018bd3b247a946d11a7 | 27 | https://gnps.ucsd.edu/ProteoSAFe/status.jsp?task=8eb61e6e07f54a5e909a1bb59285a680 | nº of nodes = 27  nº of edges = 104  nº of connected features = 25 |
|  | 50 eV | https://proteomics2.ucsd.edu/ProteoSAFe/status.jsp?task=6b33f88df7584044894d1c1ca4de3e8a | 54 | https://gnps.ucsd.edu/ProteoSAFe/status.jsp?task=4faf9d5bb3a64dcfa45165ffeac3092d | nº of nodes = 54  nº of edges = 135  nº of connected features = 44 |
|  | 70 eV | https://proteomics2.ucsd.edu/ProteoSAFe/status.jsp?task=f9c0b444353f4cd59568f2d4a3243b9d | 65 | https://gnps.ucsd.edu/ProteoSAFe/status.jsp?task=981e147b5305437b9b2b5ac544f2357c | nº of nodes = 52  nº of edges = 163  nº of connected features = 51 |
| **03**  **Neutral loss or delta mass**  (161, 100 and 161 Da) | 25 eV | https://proteomics2.ucsd.edu/ProteoSAFe/status.jsp?task=1aac8e3044294e178524cd08239e3ed0 | 150 | https://gnps.ucsd.edu/ProteoSAFe/status.jsp?task=59a1248616e74a4c8e99f8422495dc1c | nº of nodes = 49  nº of edges = 147  nº of connected features = 46 |
|  | 50 eV | https://proteomics2.ucsd.edu/ProteoSAFe/status.jsp?task=cdc3f9d8310a49f8a81172e15aba68b3 | 212 | https://gnps.ucsd.edu/ProteoSAFe/status.jsp?task=a3879705219b4a2a978cab4558aaf204 | nº of nodes = 54  nº of edges = 165  nº of connected features = 49 |
|  | 70 eV | https://proteomics2.ucsd.edu/ProteoSAFe/status.jsp?task=26fca1d436d940d7a0352e64792e66a6 | 81 | https://gnps.ucsd.edu/ProteoSAFe/status.jsp?task=0d2fe1eb73ac4d69a5264d4ac1da8b11 | nº of nodes = 32  nº of edges = 79  nº of connected features = 27 |
