# Supplementary material for: Application of feature-based molecular networking and MassQL for the MS/MS fragmentation study of depsipeptides
Source: Front Mol Biosci. 2023 Aug 1;10:1238475. doi: 10.3389/fmolb.2023.1238475 (PMC10427501; doi:10.3389/fmolb.2023.1238475)
Supplement: Supplementary file 2 [file DataSheet1.pdf]

Supplementary Material

Application of Feature-based Molecular Networking for the MS/MS fragmentation study of depsipeptides

Denise M. Selegato*, Ana C. Zanatta, Alan César Pilon, Juvenal H. Veloso, Ian Castro-Gamboa

*** Correspondence:** Denise Medeiros Selegato: denise.selegato@embl.de

1 Supplementary Data

SIRIUS4 Command for metabolite annotation

config --IsotopeSettings.filter true --FormulaSearchDB --Timeout.secondsPerTree 0 --FormulaSettings.enforced HCNOP --Timeout.secondsPerInstance 0 --AdductSettings.detectable [[M + H3N + H]+, [M + Na]+, [M - H2O + H]+, [M + H]+, [M - H4O2 + H]+] --UseHeuristic.mzToUseHeuristicOnly 650 --AlgorithmProfile qtof --IsotopeMs2Settings IGNORE --MS2MassDeviation.allowedMassDeviation 10.0ppm --NumberOfCandidatesPerIon 1 --UseHeuristic.mzToUseHeuristic 300 --FormulaSettings.detectable B,Cl,Br,Se,S --NumberOfCandidates 10 --AdductSettings.enforced , --AdductSettings.fallback [[M + Na]+, [M - H2O + H]+, [M + H]+, [M - H + Na + Na]+] --FormulaResultThreshold true --InjectElGordoCompounds false --StructureSearchDB BIO,METACYC,CHEBI,COCONUT,ECOCYCMINE,GNPS,HMDB,HSDB,KEGG,KEGGMINE,KNAPSACK,MACONDA,MESH,NORMAN,UNDP,PLANTCYC,PUBCHEM,PUBMED,YMDB,YMDBMINE,ZINCBIO --RecomputeResults false formula fingerprint structure.

Selection of tolerance used in FBMN and MassQL

The tolerance used for feature-based Molecular Networking and MassQL were based on non-strict tolerance levels recommended for QToF instruments. Moreover, the precursor ion mass tolerance is set at lower tolerance than MS/MS because beauvericin identifications were improved when spectra were searched with a wide mass tolerance window and precursor mass is used as a filter to discard incorrect matches.

MassQL Queries

Query 01

MassQL Query

QUERY scaninfo(MS2DATA) WHERE MS2PROD=(362.2 OR 262.1 OR 244.1 OR 134.1):TOLERANCEMZ=0.1

|||

QUERY scaninfo(MS2DATA) WHERE MS2PROD=(384.2 OR 284.1 OR 266.1):TOLERANCEMZ=0.1

Query Parse Visualization

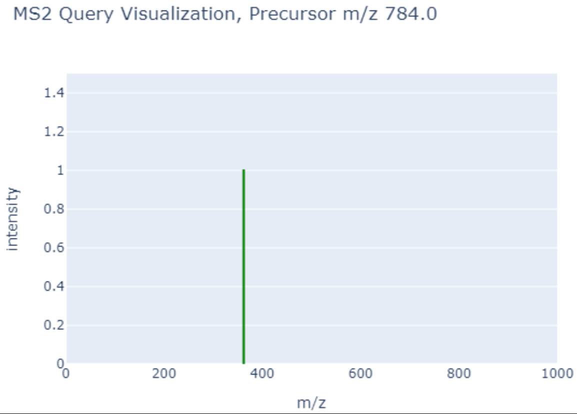

Query 02

MassQL Query

QUERY scaninfo (MS2DATA) WHERE MS2PROD=362.2: TOLERANCEMZ=0.1 AND MS2PROD=262.1: TOLERANCEMZ=0.1 AND MS2PROD=262.1 – formula (H2O): TOLERANCEMZ=0.1 AND MS2PROD=134.1: TOLERANCEMZ=0.1

|||

QUERY scaninfo (MS2DATA) WHERE MS2PROD=384.2: TOLERANCEMZ=0.1 AND MS2PROD=284.1: TOLERANCEMZ=0.1 AND MS2PROD=284.1 – formula (H2O): TOLERANCEMZ=0.1

Query Parse Visualization

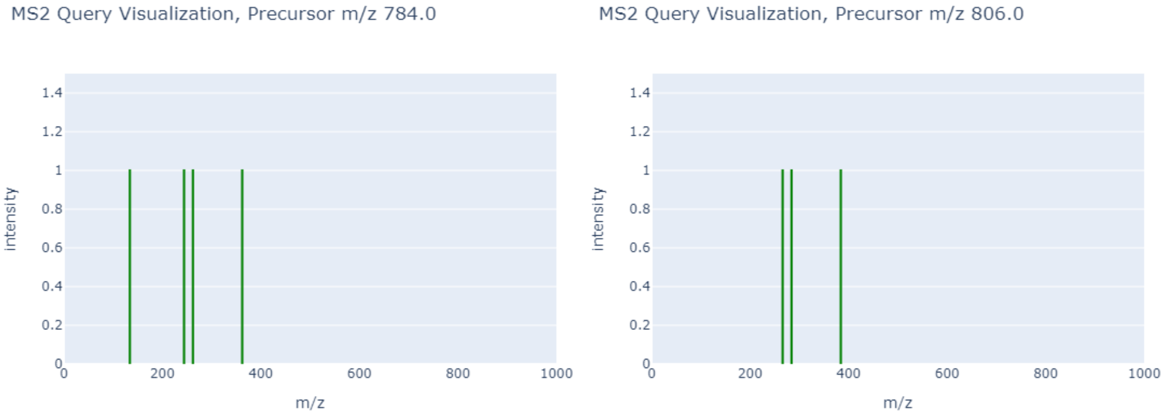

Query 03
**MassQL Query**
QUERY scaninfo(MS2DATA) WHERE MS2PROD=X AND MS2PROD=X+100.1: TOLERANCEMZ=0.1: INTENSITYPERCENT=1 AND MS2PROD=X+261.1: TOLERANCEMZ=0.1: INTENSITYPERCENT=1
**Query Parse Visualization**
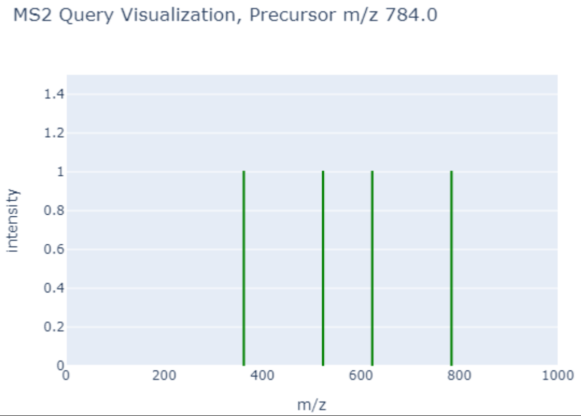

2 Supplementary Figures and Tables

2.1 Supplementary Figures

(A)

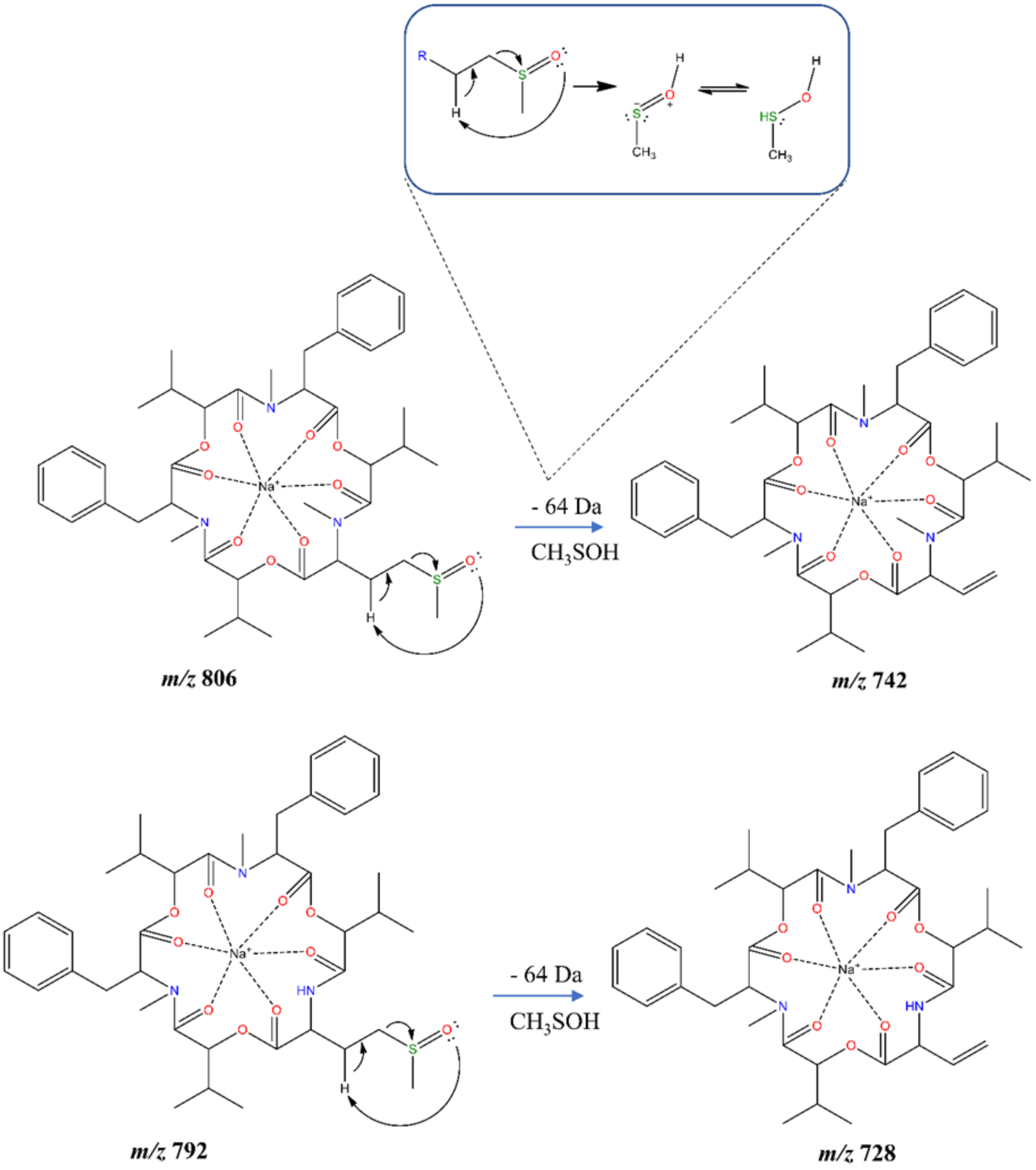

(B)

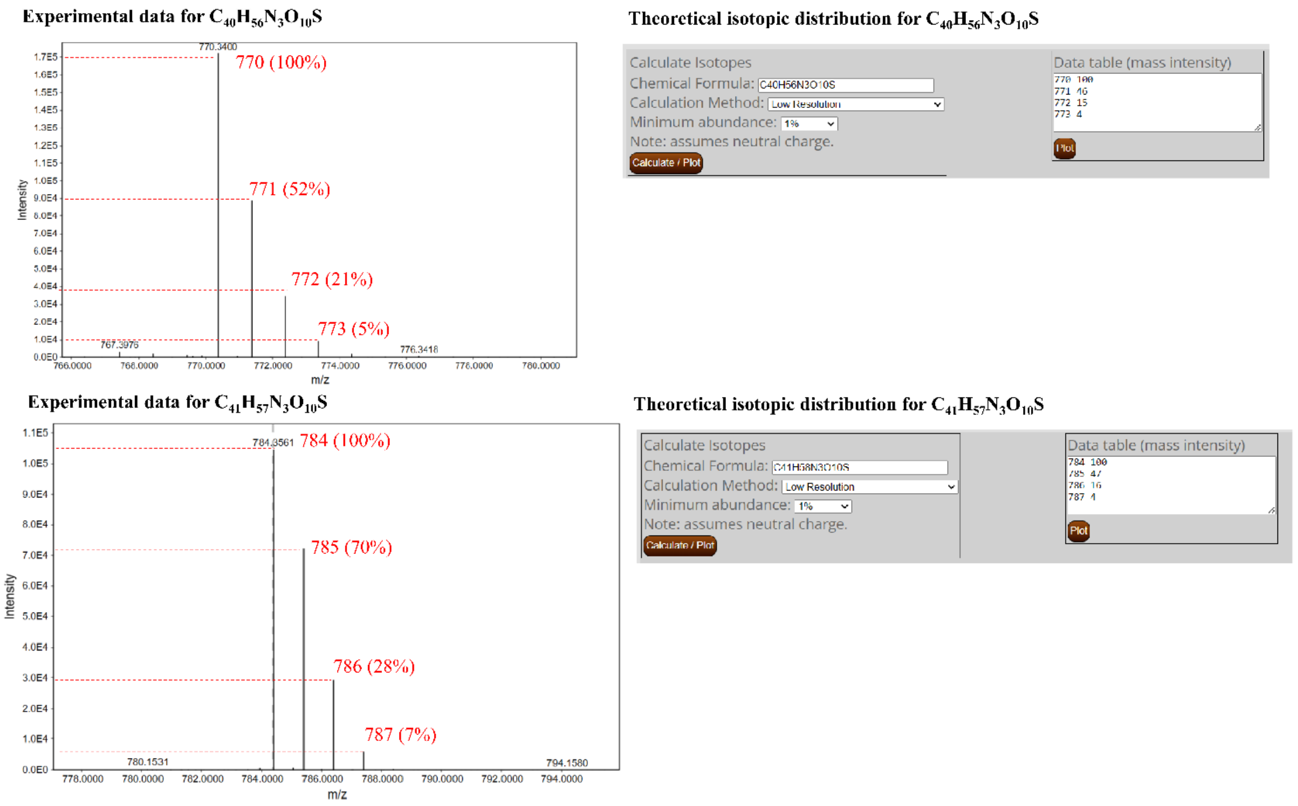

Supplementary Figure 1. (A) The rearrangement mechanism for the neutral loss of 64 Da in the methionine sulfoxide residue. (B) Comparison between theoretical and experimental isotopic distribution of compounds 1 and 2.

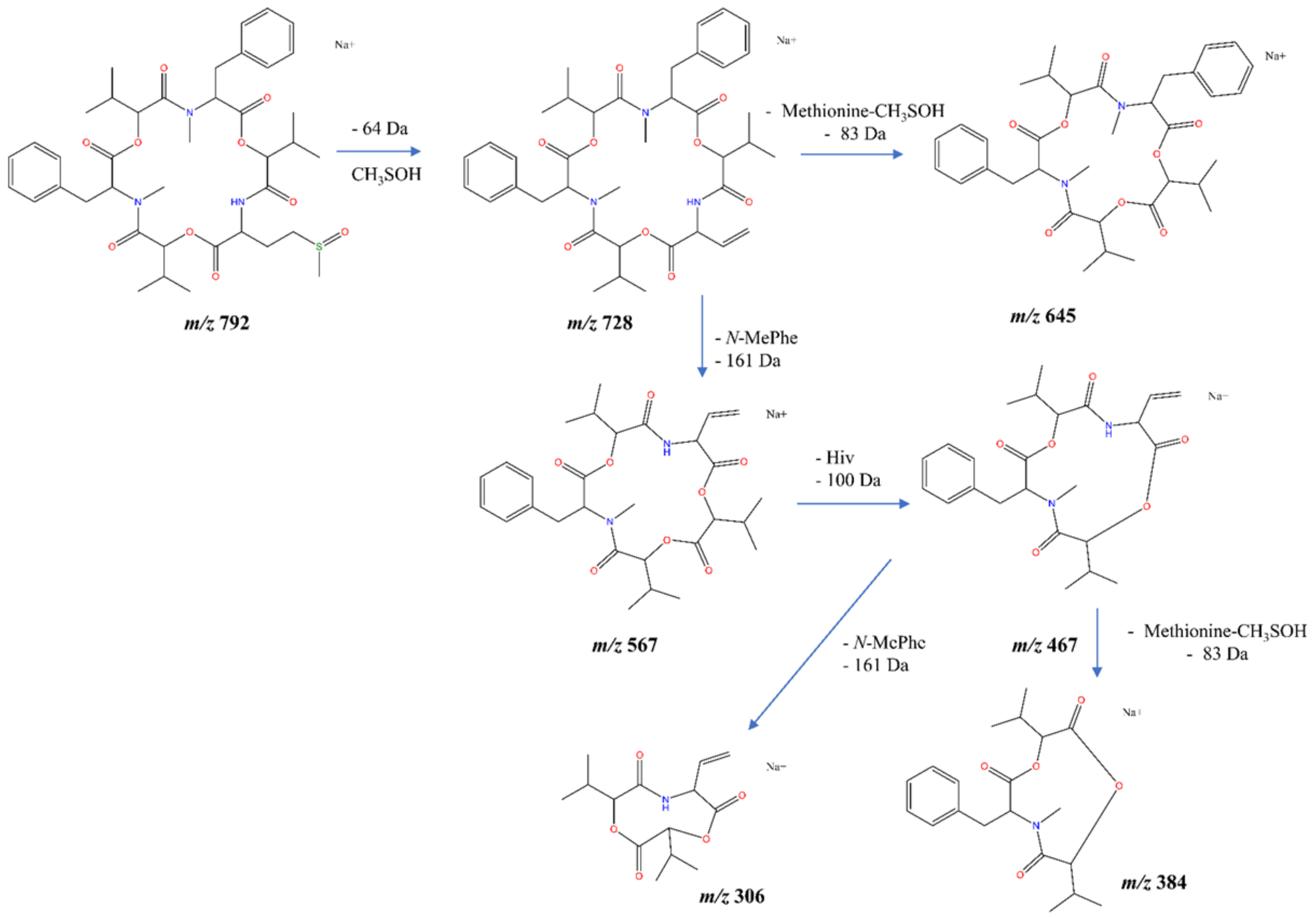

Supplementary Figure 2. ESI-MS/MS fragmentation scheme for *m/z* 792.3536 $[M+Na]^+$, RT 30.37 minutes. Proposed molecular formula: $C_{40}H_{55}N_3O_{10}S$ (mass error of 3 ppm).

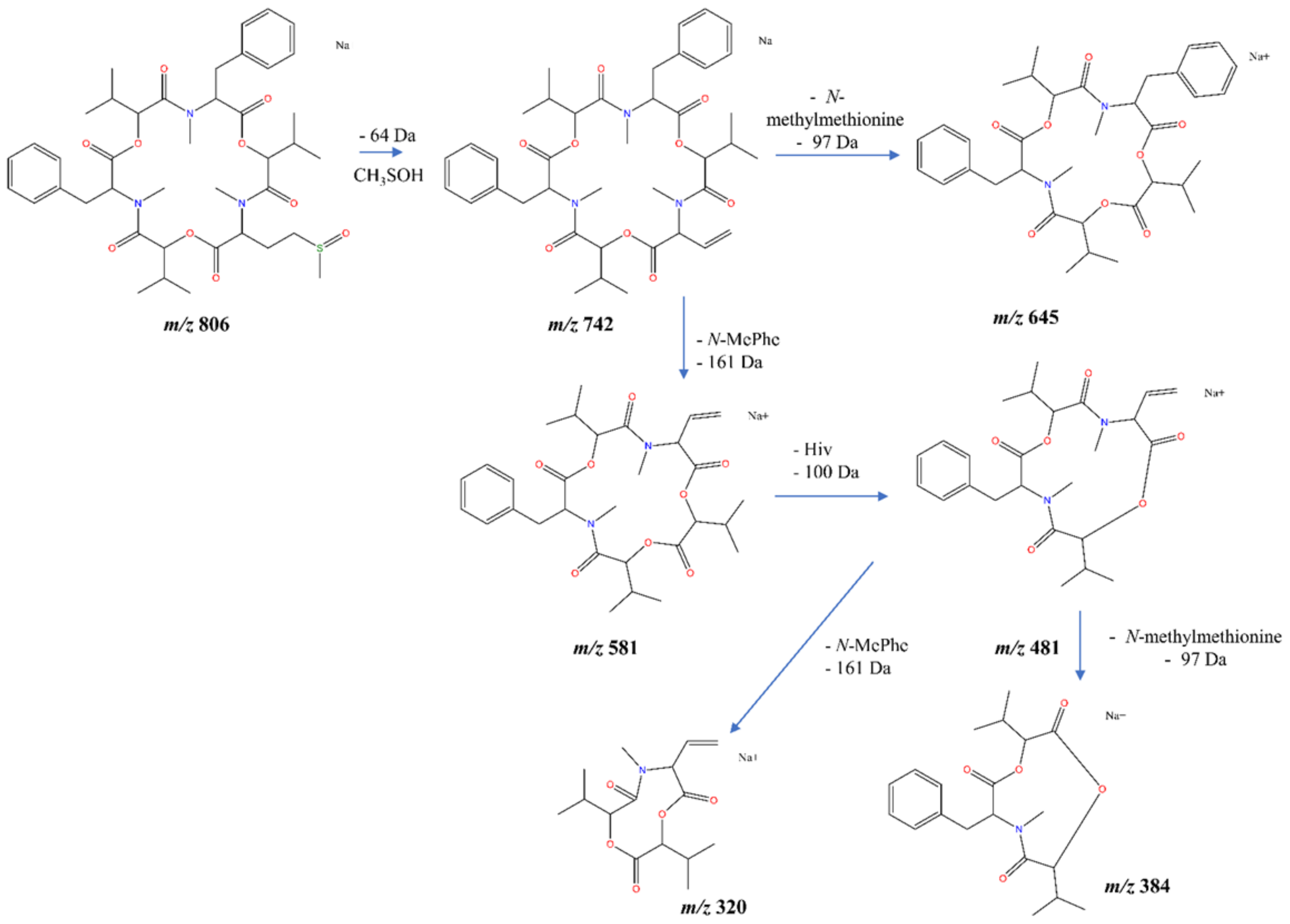

Supplementary Figure 3. ESI-MS/MS Fragmentation scheme for *m/z* 806.3695 $[M+Na]^+$, RT 30.62 minutes. Proposed molecular formula: $C_{41}H_{57}N_3O_{10}S$ (mass error of 3 ppm).

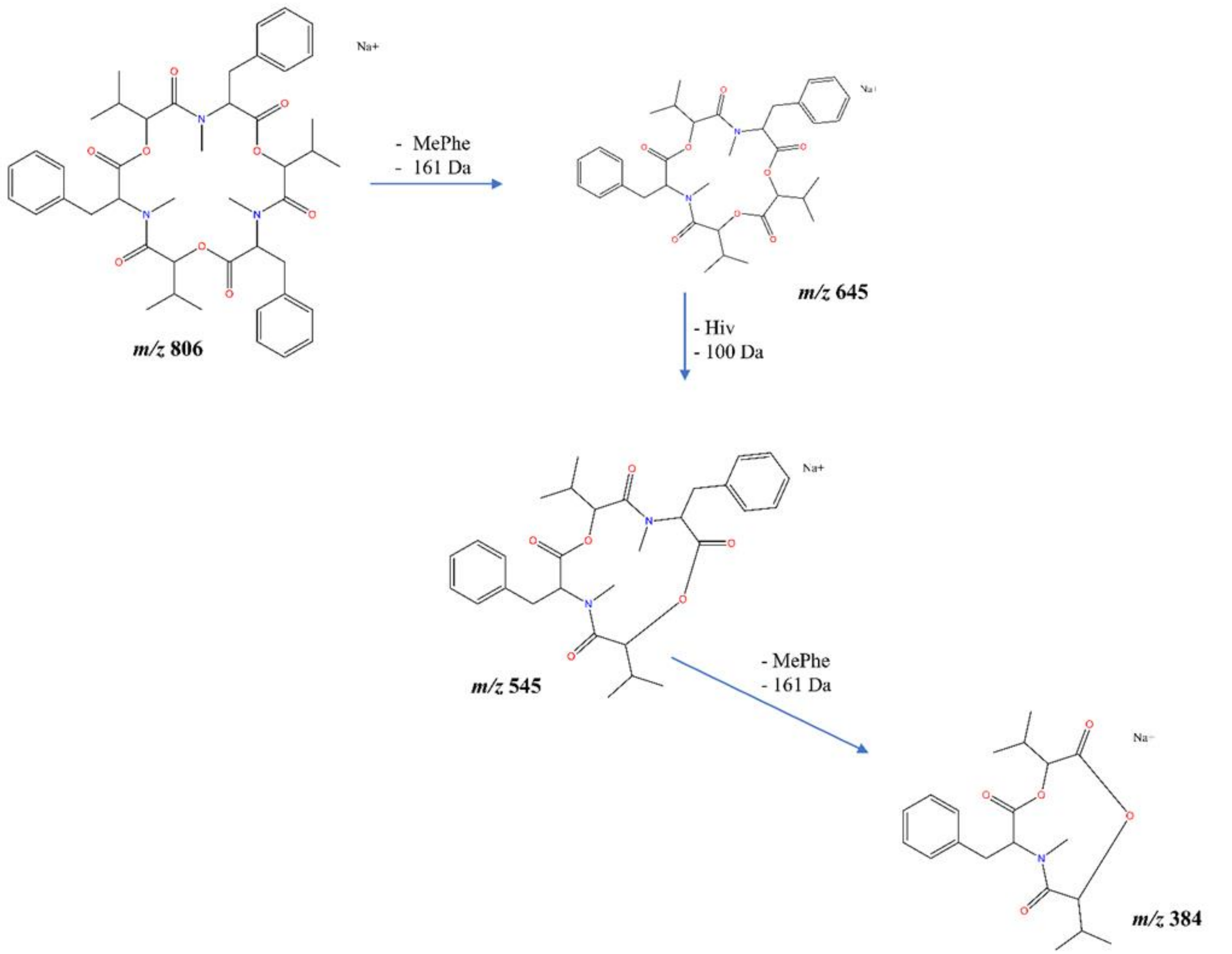

Supplementary Figure 4. ESI-MS/MS Fragmentation scheme for *m/z* 806.4031 $[M+Na]^+$, RT 38.50 minutes. Proposed molecular formula: $C_{45}H_{57}N_3O_9$ (mass error of 4.83 ppm).

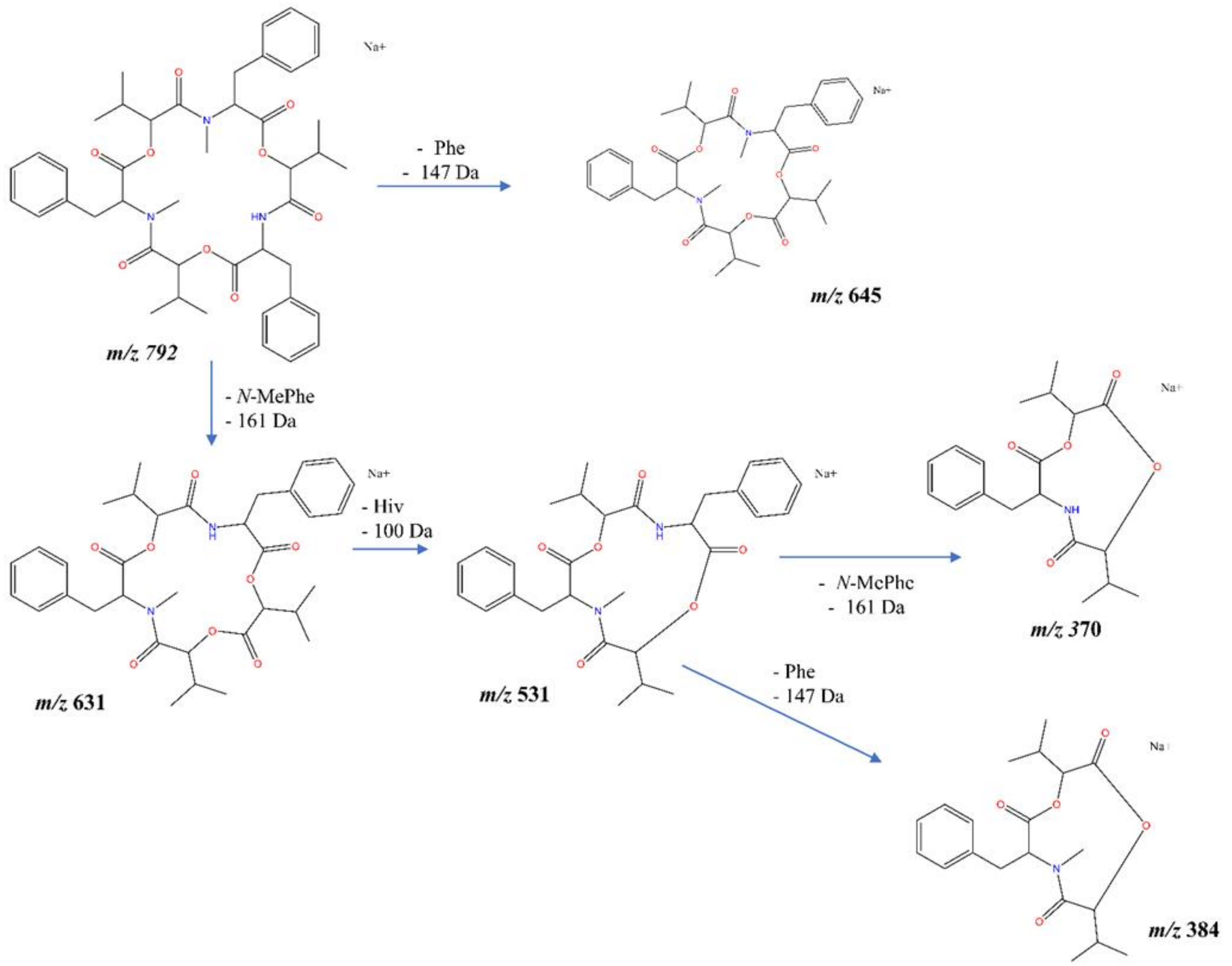

Supplementary Figure 5. ESI-MS/MS Fragmentation scheme for *m/z* 792.3874 [M+Na]$^+$, RT 37.2 minutes. Proposed molecular formula: $C_{44}H_{55}N_3O_9$ (mass error of 4.79 ppm).

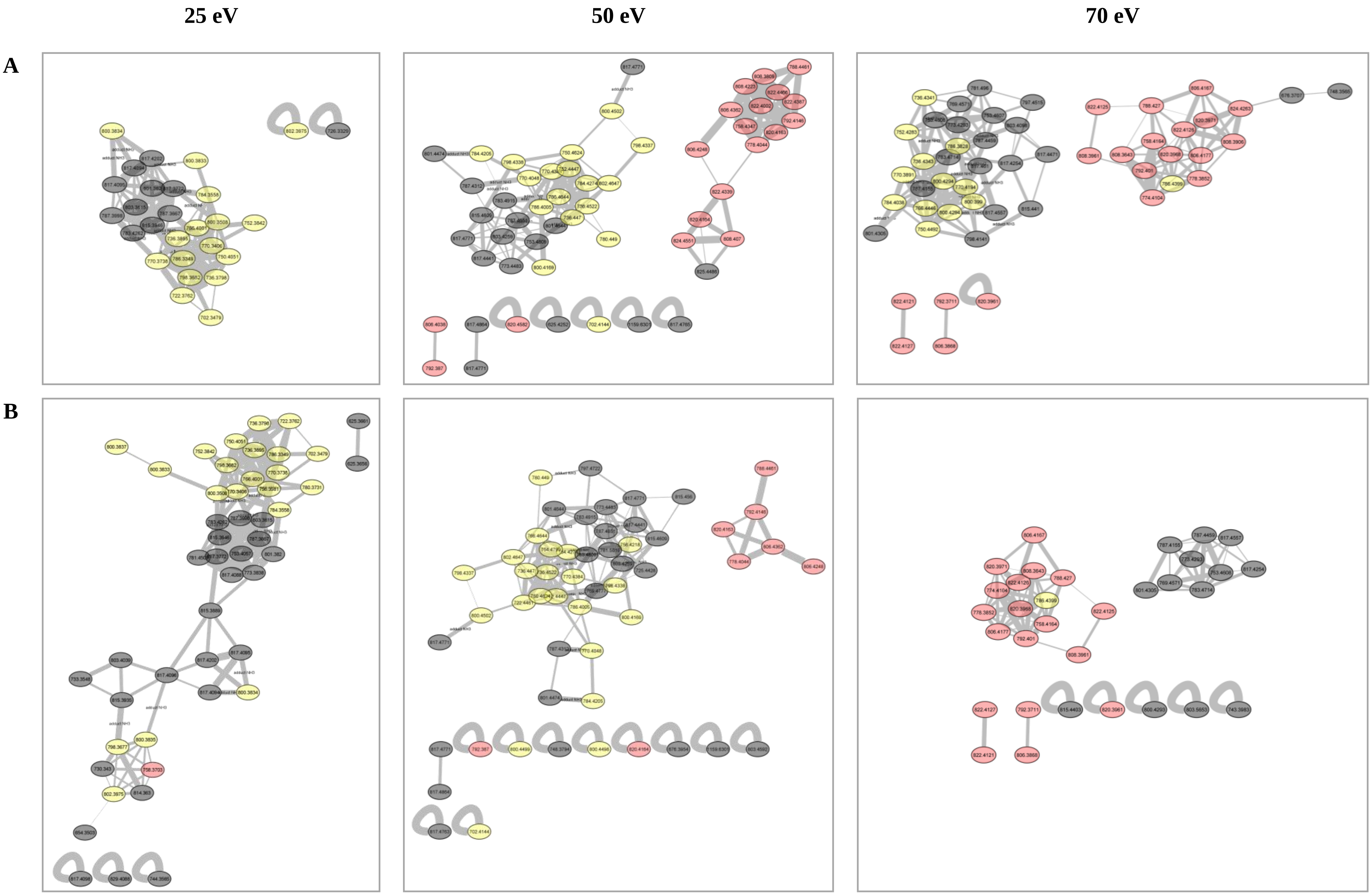

Supplementary Figure 6. MassQL-FBMN clusters at 25, 50, and 70 eV for **(A)** query 2 and **(B)** query 3. Protonated precursor ions are colored in yellow and sodiated ions colored in red.

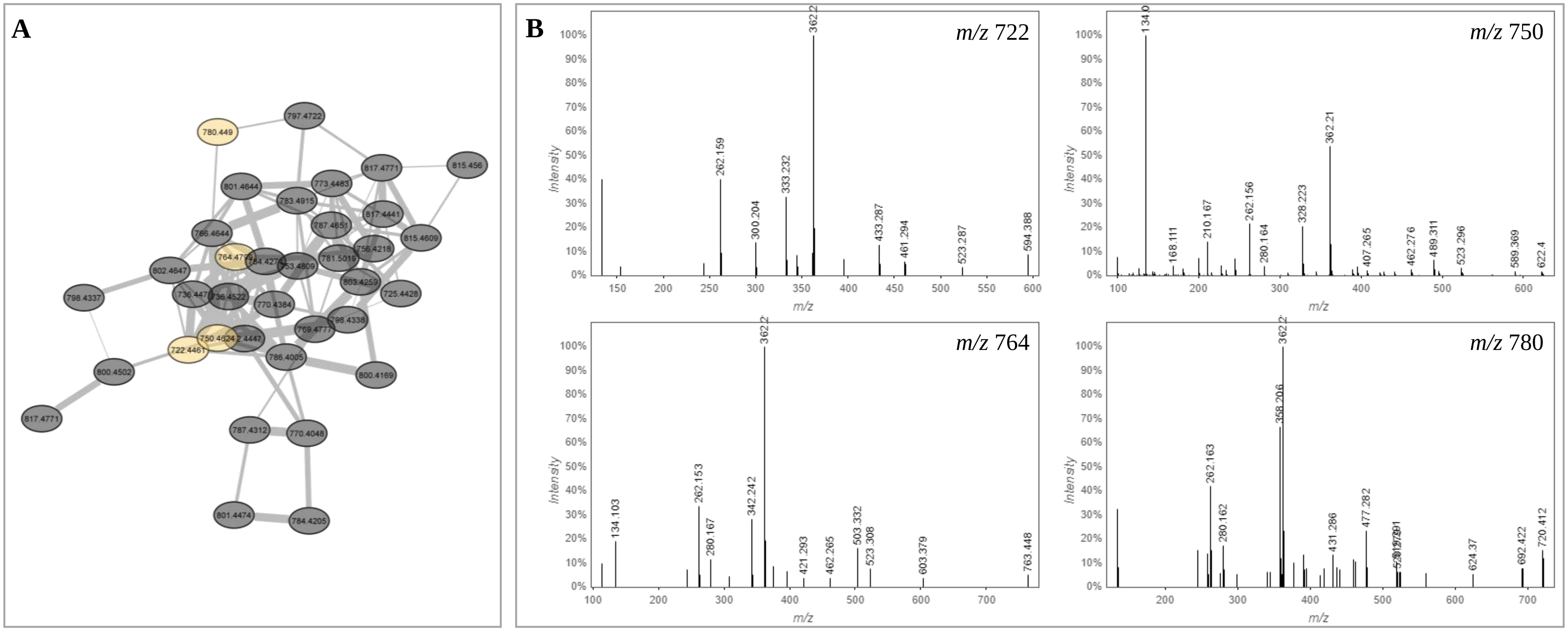

Supplementary Figure 7. (A) Protonated beauvericin cluster from the MassQL-FBMN at 50 eV. Nodes colored in grey are found in all CIDs whereas nodes colored in yellow are specific for only protonated clusters. **(B)** MS/MS spectra of the precursor ions at *m/z* 722, *m/z* 750, *m/z* 764, and *m/z* 780 at collision energy of 50 eV.

2.2 Supplementary Tables

Supplementary Table 1. Data availability.

Type of Data	Platform	Link
Raw Data	**MassIVE**	https://massive.ucsd.edu/ProteoSAFe/dataset.jsp?accession=MSV000091616<br><br>ftp://massive.ucsd.edu/MSV000091616
LC-MS/MS data processing and batch	**MassIVE**	https://massive.ucsd.edu/ProteoSAFe/dataset.jsp?accession=MSV000091616<br><br>ftp://massive.ucsd.edu/MSV000091616
FBMN 70 eV	**GNPS**	https://gnps.ucsd.edu/ProteoSAFe/status.jsp?task=28563ce9301b47ed8e042548567fb38f
FBMN 50 eV	**GNPS**	https://gnps.ucsd.edu/ProteoSAFe/status.jsp?task=941df39105424f95a5bd0efbe2549315
FBMN 25 eV	**GNPS**	https://gnps.ucsd.edu/ProteoSAFe/status.jsp?task=9e914444b87b44d79c9b7e00b949a7bb

Supplementary Table 2. MassQL queries using product ion formation (Queries 1 and 2) and neutral losses (Query 3).

Query	Collision energy	Query Link	nº of scans detected	FBMN link	Networking statistics
**01 Product ion formation** (*m/z* 362 OR 262 OR 244 OR 134| *m/z* 384 OR 284 OR 266)	25 eV	https://proteomics2.ucsd.edu/ProteoSAFe/status.jsp?task=1b07b2246fc540f69a41ed2844645ed3	112	-	-
	50 eV	https://proteomics2.ucsd.edu/ProteoSAFe/status.jsp?task=a8a3cc24289841a38db7e18fa8d44b70	167	-	-
	70 eV	https://proteomics2.ucsd.edu/ProteoSAFe/status.jsp?task=0d0235a8c276424dbf10c5ab40a3dbcc	156	-	-
**02 Product ion formation** (*m/z* 362 AND 262 AND 244 AND 134| *m/z* 384 AND 284 AND 266)	25 eV	https://proteomics2.ucsd.edu/ProteoSAFe/status.jsp?task=81b601d243284018bd3b247a946d11a7	27	https://gnps.ucsd.edu/ProteoSAFe/status.jsp?task=8eb61e6e07f54a5e909a1bb59285a680	nº of nodes = 27<br>nº of edges = 104<br>nº of connected features = 25
	50 eV	https://proteomics2.ucsd.edu/ProteoSAFe/status.jsp?task=6b33f88df7584044894d1c1ca4de3e8a	54	https://gnps.ucsd.edu/ProteoSAFe/status.jsp?task=4faf9d5bb3a64dcfa45165ffeac3092d	nº of nodes = 54<br>nº of edges = 135<br>nº of connected features = 44
	70 eV	https://proteomics2.ucsd.edu/ProteoSAFe/status.jsp?task=f9c0b444353f4cd59568f2d4a3243b9d	65	https://gnps.ucsd.edu/ProteoSAFe/status.jsp?task=981e147b5305437b9b2b5ac544f2357c	nº of nodes = 52<br>nº of edges = 163<br>nº of connected features = 51
**03 Neutral loss or delta mass** (161, 100 and 161 Da)	25 eV	https://proteomics2.ucsd.edu/ProteoSAFe/status.jsp?task=1aac8e3044294e178524cd08239e3ed0	150	https://gnps.ucsd.edu/ProteoSAFe/status.jsp?task=59a1248616e74a4c8e99f8422495dc1c	nº of nodes = 49<br>nº of edges = 147<br>nº of connected features = 46
	50 eV	https://proteomics2.ucsd.edu/ProteoSAFe/status.jsp?task=cdc3f9d8310a49f8a81172e15aba68b3	212	https://gnps.ucsd.edu/ProteoSAFe/status.jsp?task=a3879705219b4a2a978cab4558aaf204	nº of nodes = 54<br>nº of edges = 165<br>nº of connected features = 49
	70 eV	https://proteomics2.ucsd.edu/ProteoSAFe/status.jsp?task=26fca1d436d940d7a0352e64792e66a6	81	https://gnps.ucsd.edu/ProteoSAFe/status.jsp?task=0d2fe1eb73ac4d69a5264d4ac1da8b11	nº of nodes = 32<br>nº of edges = 79<br>nº of connected features = 27
